# Supplementary figures and images for: Histone variants shape chromatin states in Arabidopsis
Source: eLife. 2023 Jul 19;12:RP87714. doi: 10.7554/eLife.87714 (PMC10393023; doi:10.7554/eLife.87714)

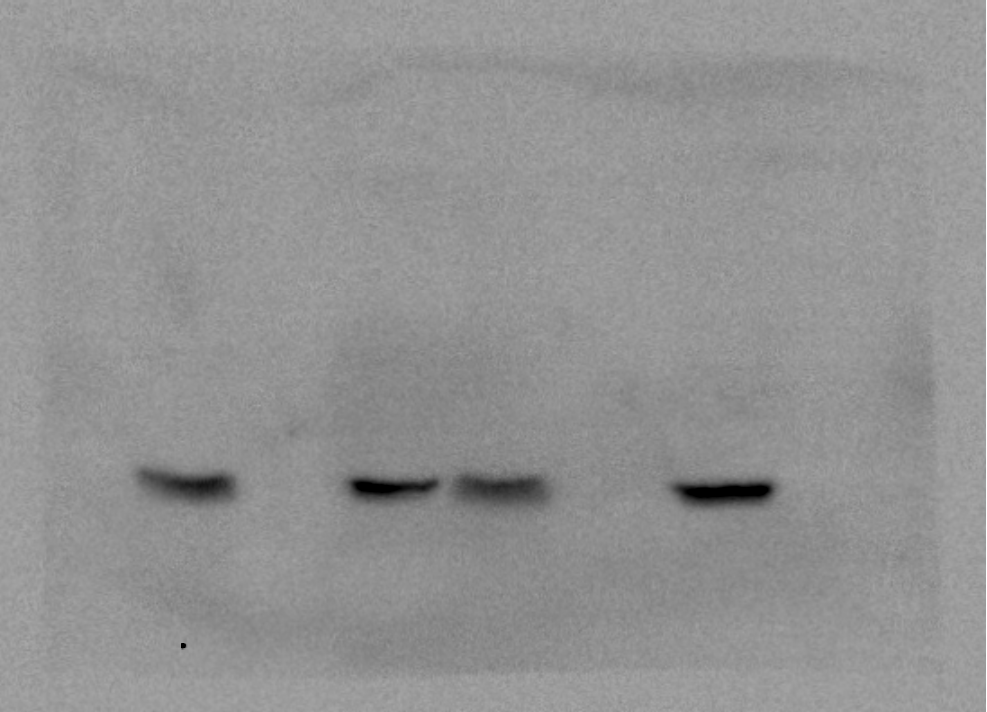

Supplement: Figure 1—source data 1. [file elife-87714-fig1-data1.zip › Figure 1-Source Data 1/Figure 1B Western blots/WB H2A.13.tif]

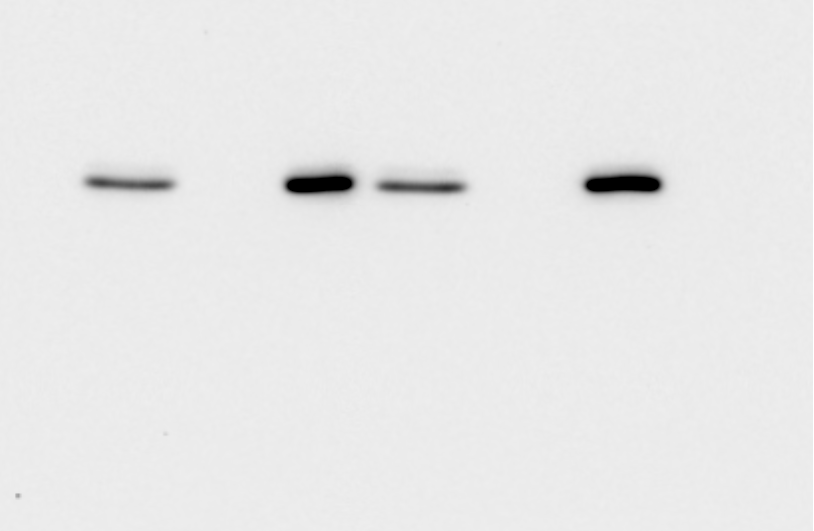

Supplement: Figure 1—source data 1. [file elife-87714-fig1-data1.zip › Figure 1-Source Data 1/Figure 1B Western blots/WB H2A.W.6.tif]

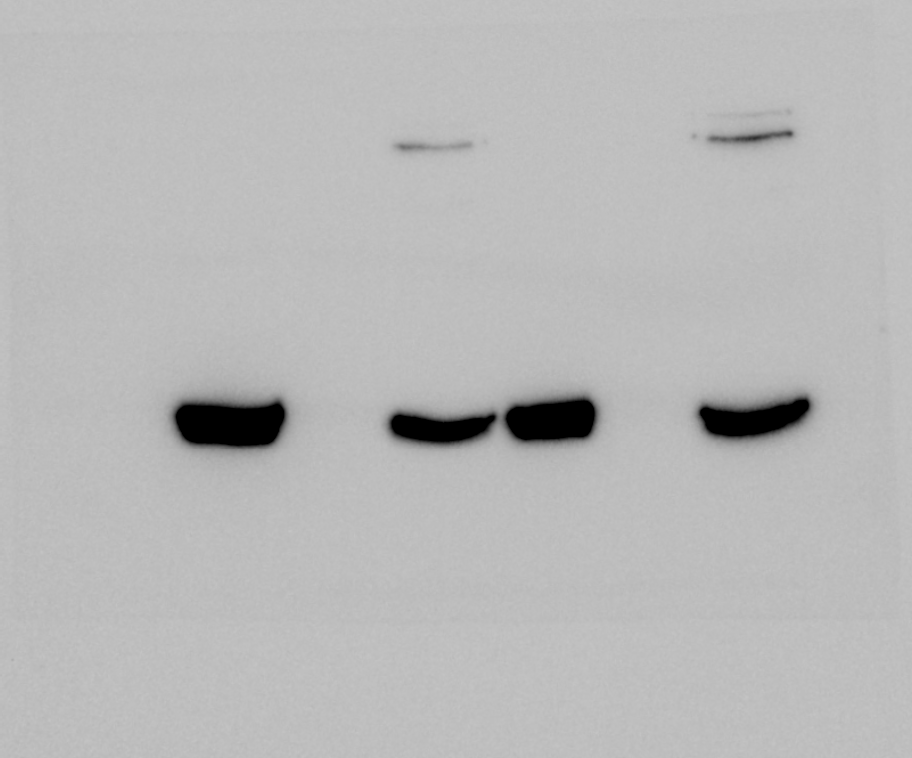

Supplement: Figure 1—source data 1. [file elife-87714-fig1-data1.zip › Figure 1-Source Data 1/Figure 1B Western blots/WB H2A.W.7.tif]

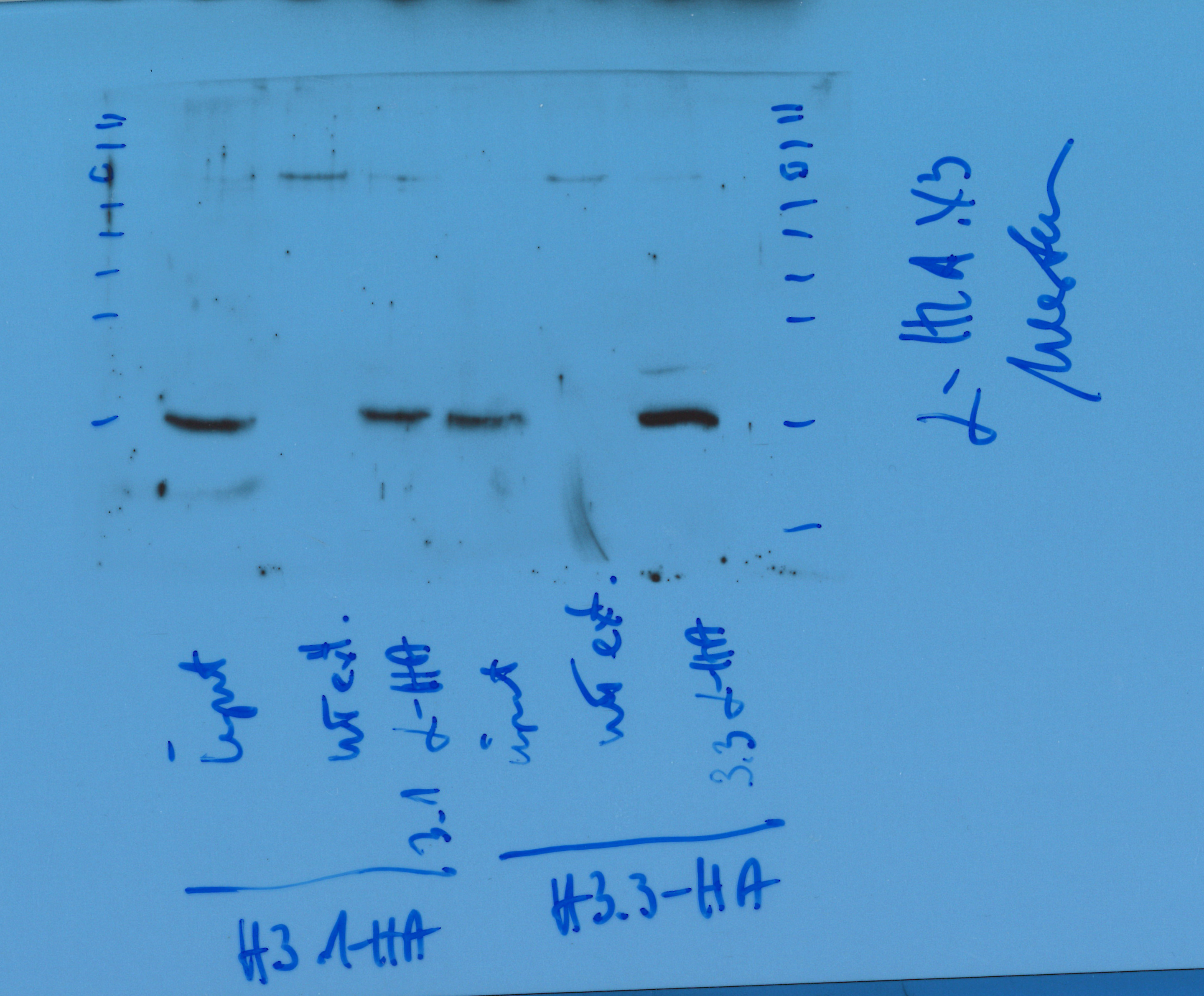

Supplement: Figure 1—source data 1. [file elife-87714-fig1-data1.zip › Figure 1-Source Data 1/Figure 1B Western blots/WB H2A.X.tif]

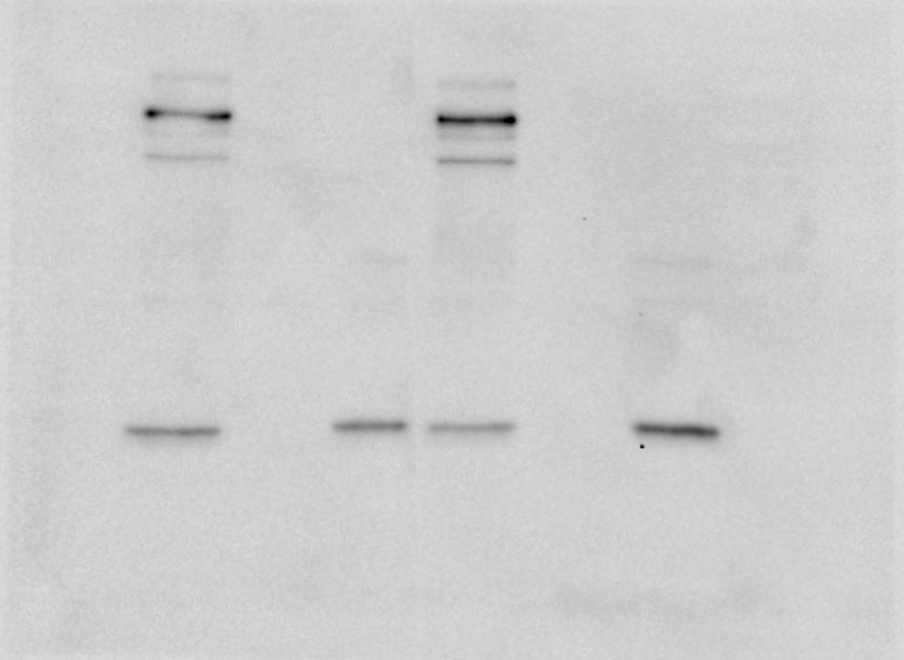

Supplement: Figure 1—source data 1. [file elife-87714-fig1-data1.zip › Figure 1-Source Data 1/Figure 1B Western blots/WB H2A.Z.9.tif]

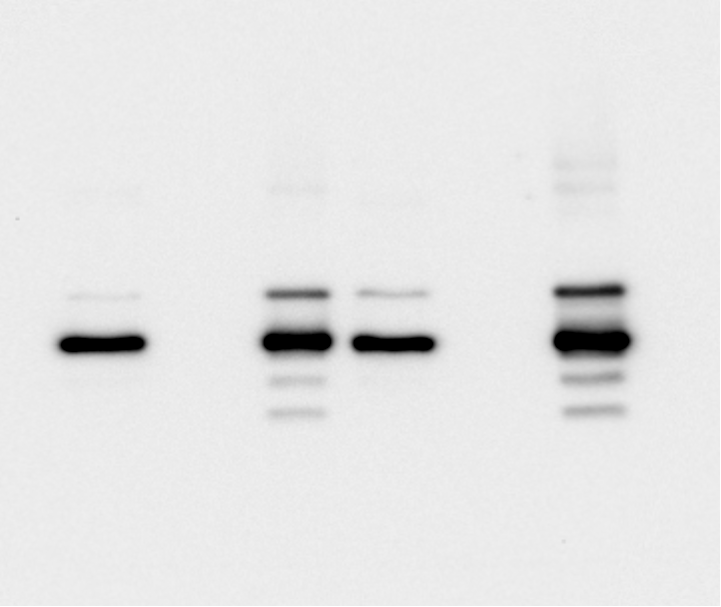

Supplement: Figure 1—source data 1. [file elife-87714-fig1-data1.zip › Figure 1-Source Data 1/Figure 1B Western blots/WB H3.tif]

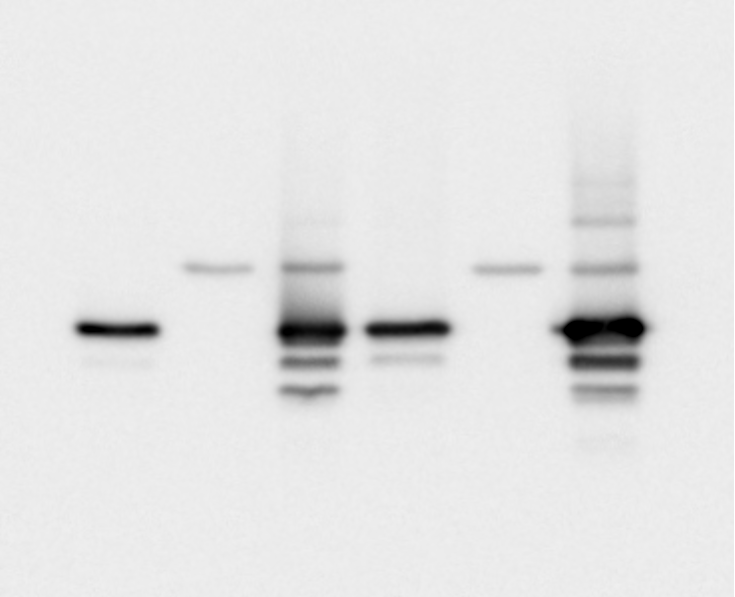

Supplement: Figure 1—source data 1. [file elife-87714-fig1-data1.zip › Figure 1-Source Data 1/Figure 1B Western blots/WB HA.tif]

Figure 1B

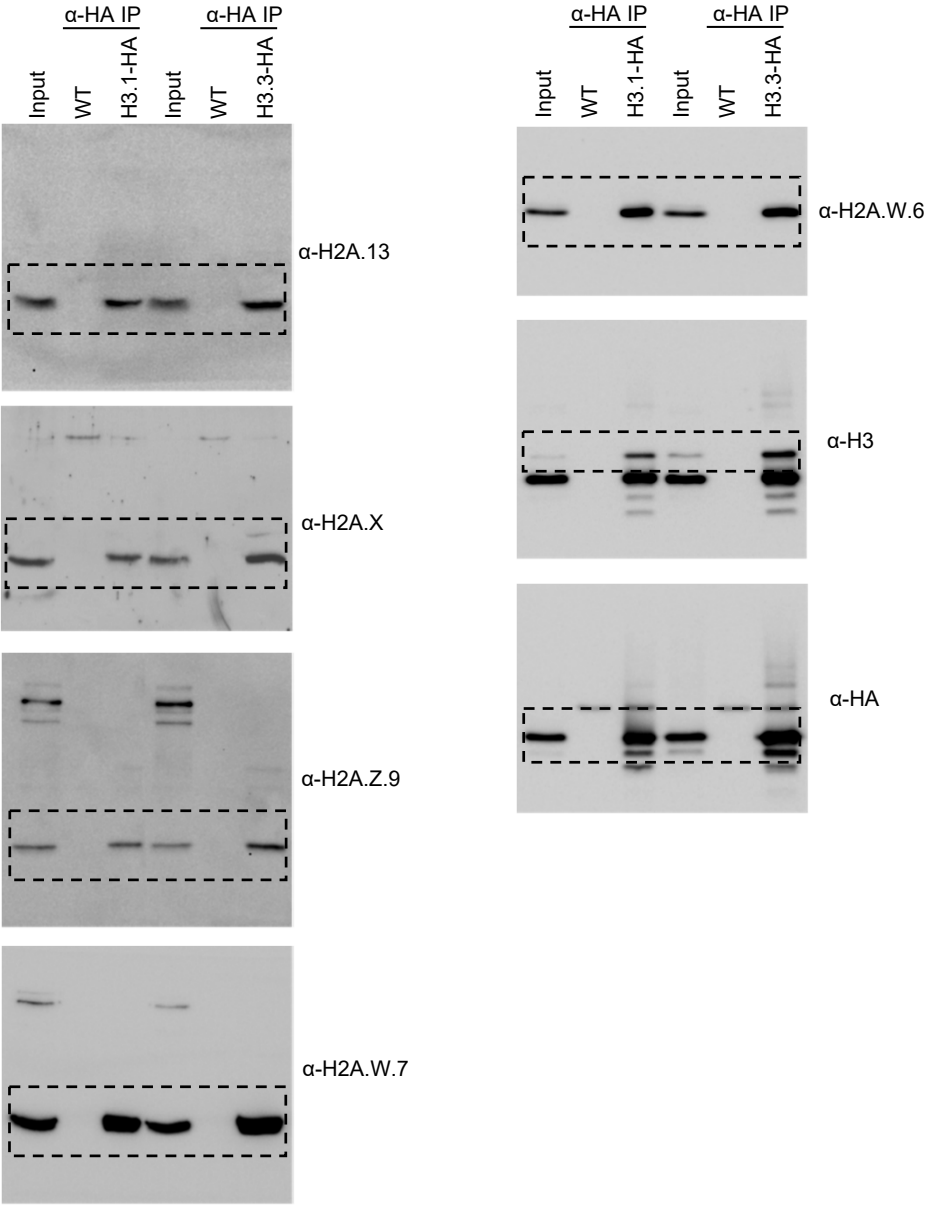

Supplement: Figure 1—source data 1. [file elife-87714-fig1-data1.zip › Figure 1-Source Data 1/WB data Figure 1B.pdf]

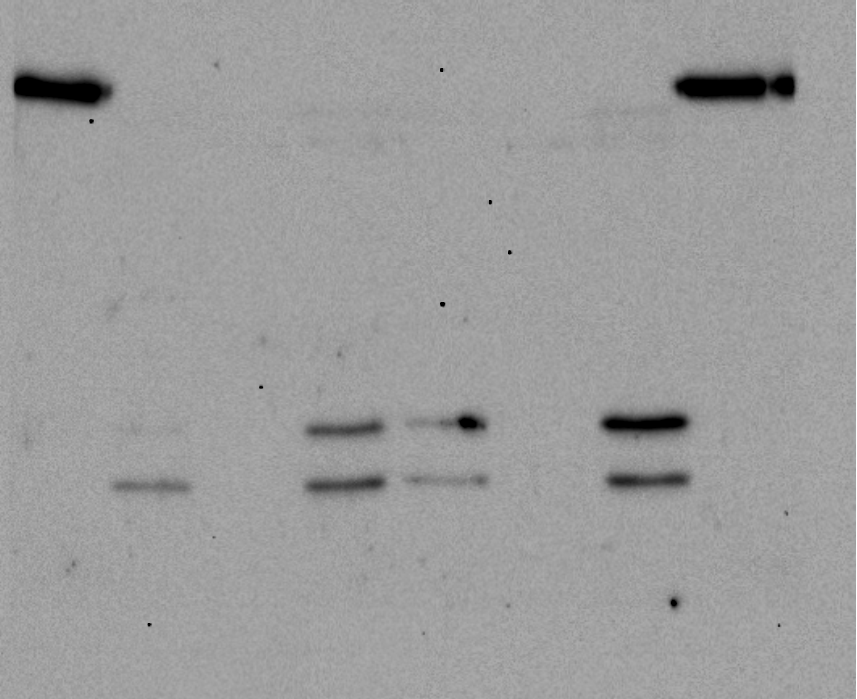

Supplement: Figure 1—source data 2. [file elife-87714-fig1-data2.zip › Figure 1-Source Data 2/Figure 1C Western blots/WB H3K122ac.tif]

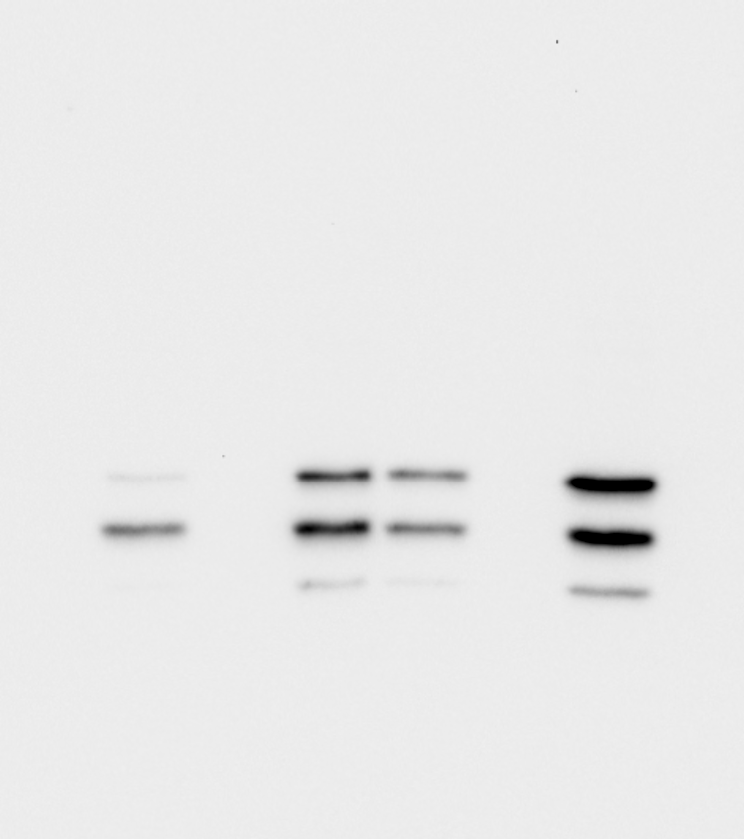

Supplement: Figure 1—source data 2. [file elife-87714-fig1-data2.zip › Figure 1-Source Data 2/Figure 1C Western blots/WB H3K27ac.tif]

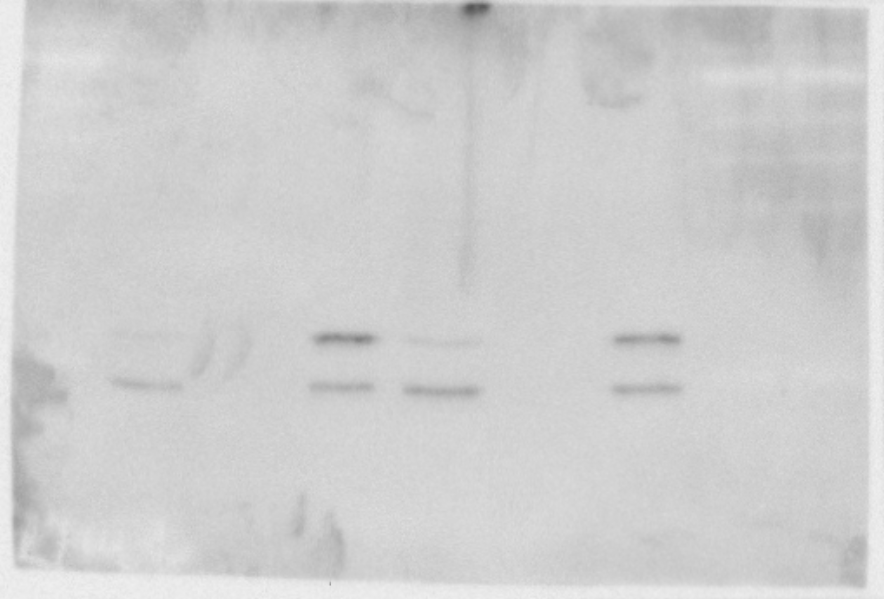

Supplement: Figure 1—source data 2. [file elife-87714-fig1-data2.zip › Figure 1-Source Data 2/Figure 1C Western blots/WB H3K27me1.tif]

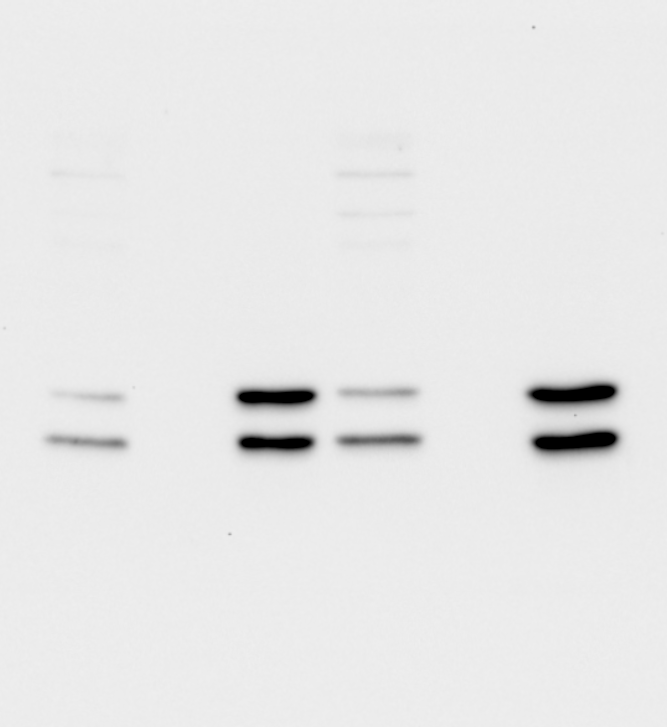

Supplement: Figure 1—source data 2. [file elife-87714-fig1-data2.zip › Figure 1-Source Data 2/Figure 1C Western blots/WB H3K27me2.tif]

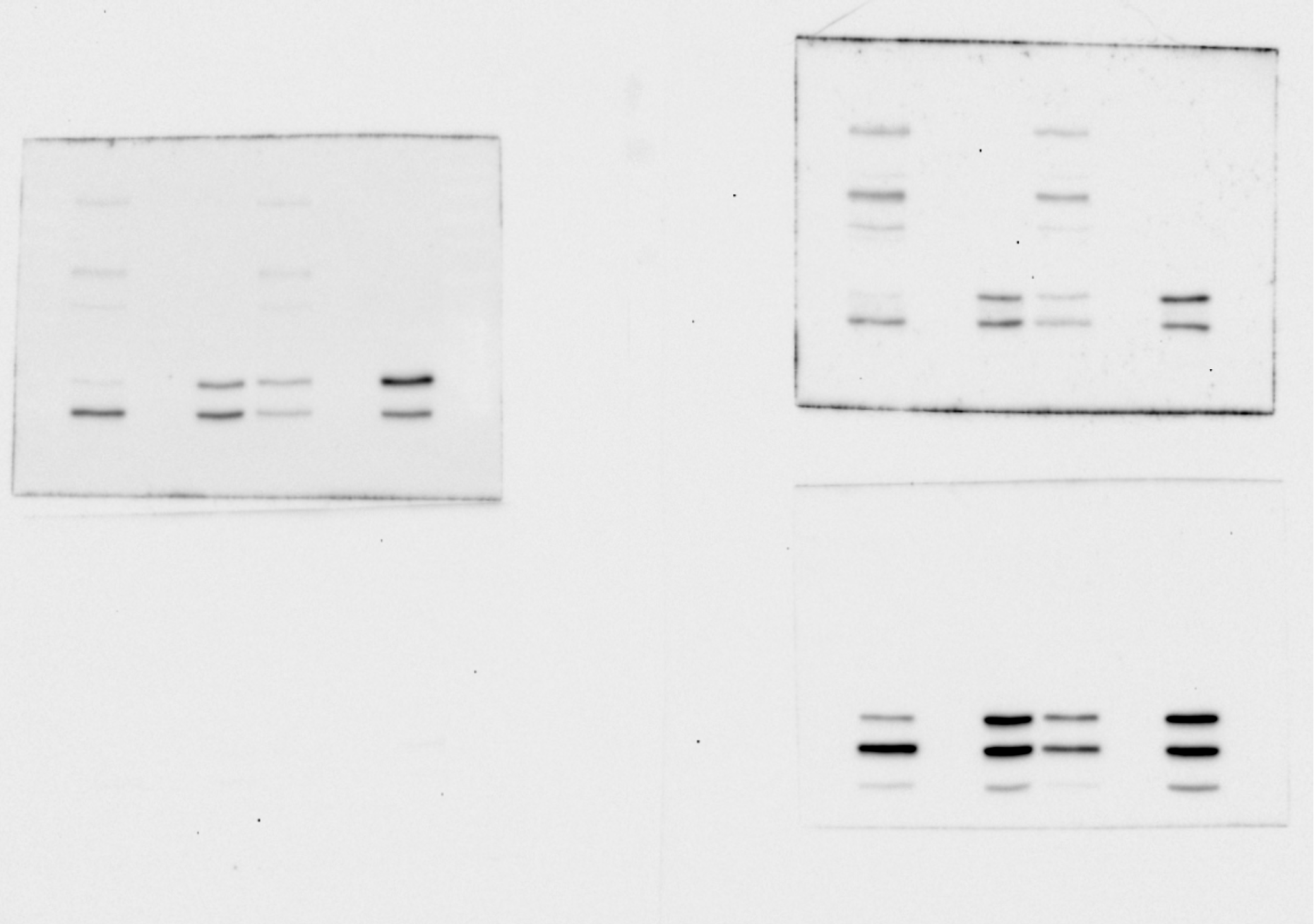

Supplement: Figure 1—source data 2. [file elife-87714-fig1-data2.zip › Figure 1-Source Data 2/Figure 1C Western blots/WB H3K36me1_H3K23ac_H3K18ac.tif]

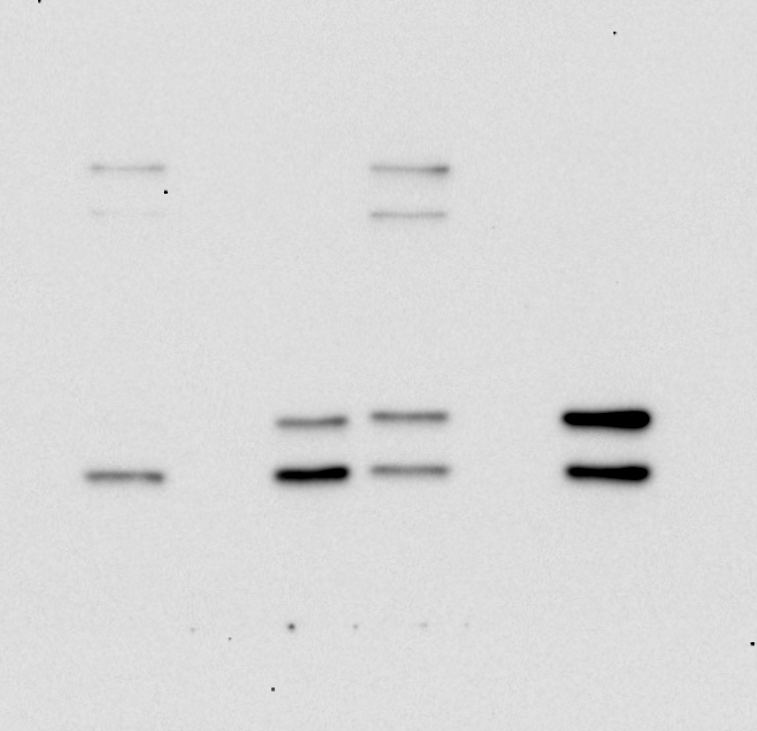

Supplement: Figure 1—source data 2. [file elife-87714-fig1-data2.zip › Figure 1-Source Data 2/Figure 1C Western blots/WB H3K36me2.tif]

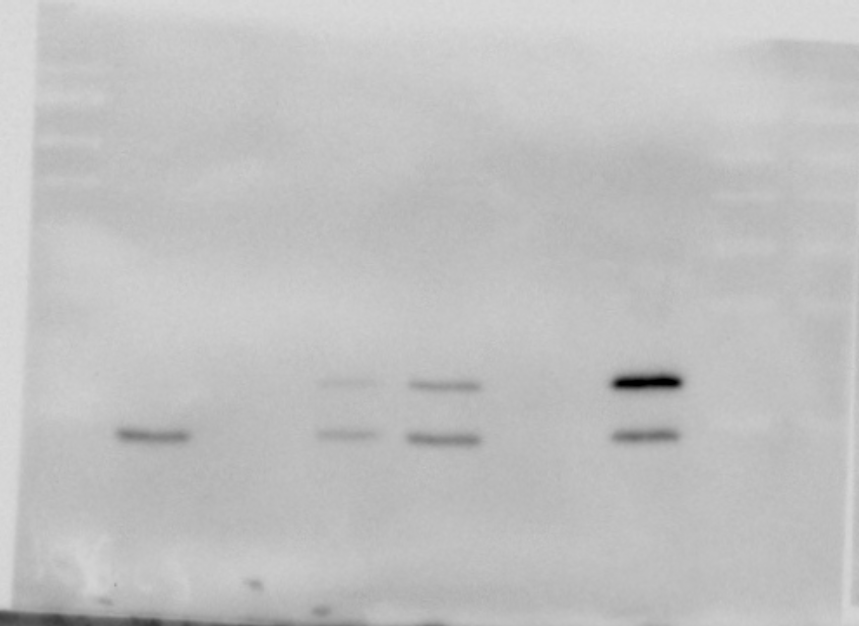

Supplement: Figure 1—source data 2. [file elife-87714-fig1-data2.zip › Figure 1-Source Data 2/Figure 1C Western blots/WB H3K36me3.tif]

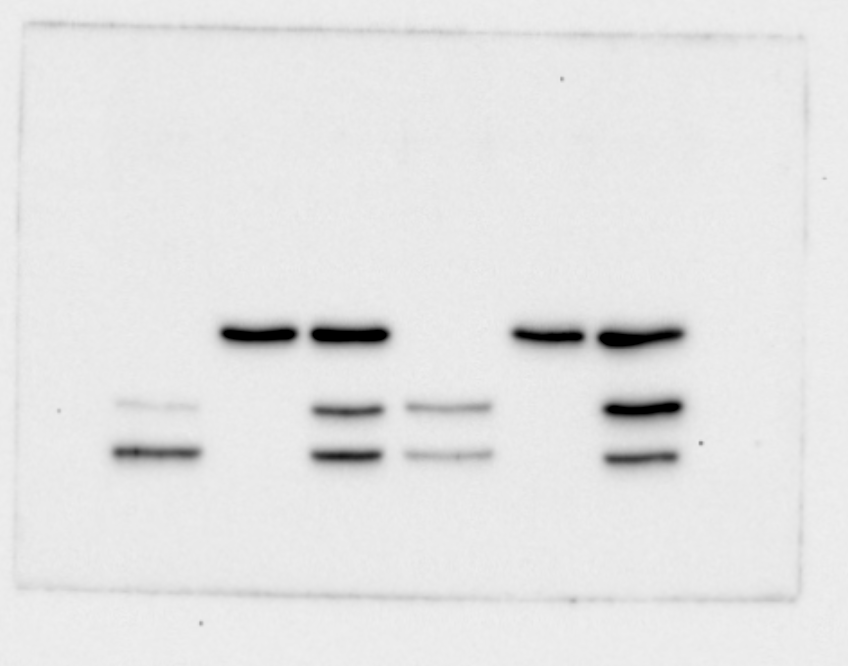

Supplement: Figure 1—source data 2. [file elife-87714-fig1-data2.zip › Figure 1-Source Data 2/Figure 1C Western blots/WB H3K4me1.tif]

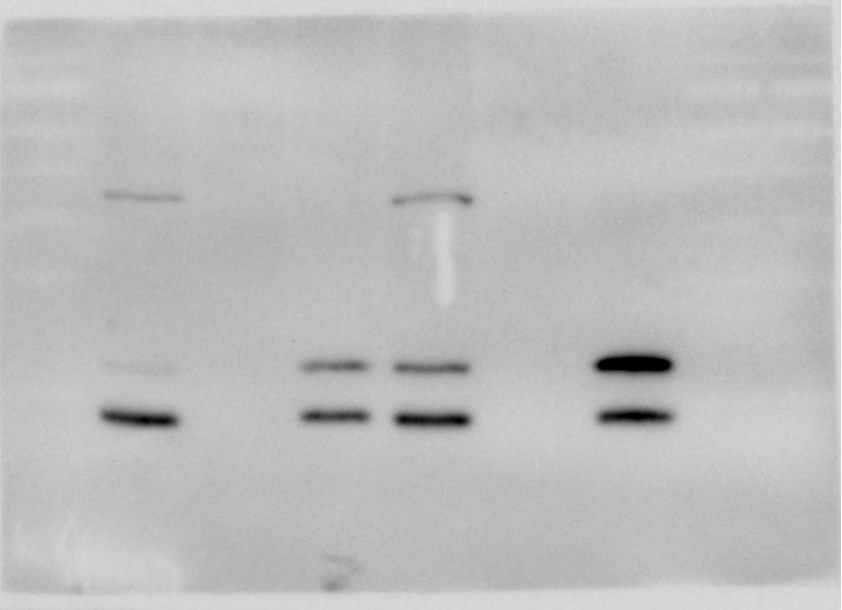

Supplement: Figure 1—source data 2. [file elife-87714-fig1-data2.zip › Figure 1-Source Data 2/Figure 1C Western blots/WB H3K4me3.tif]

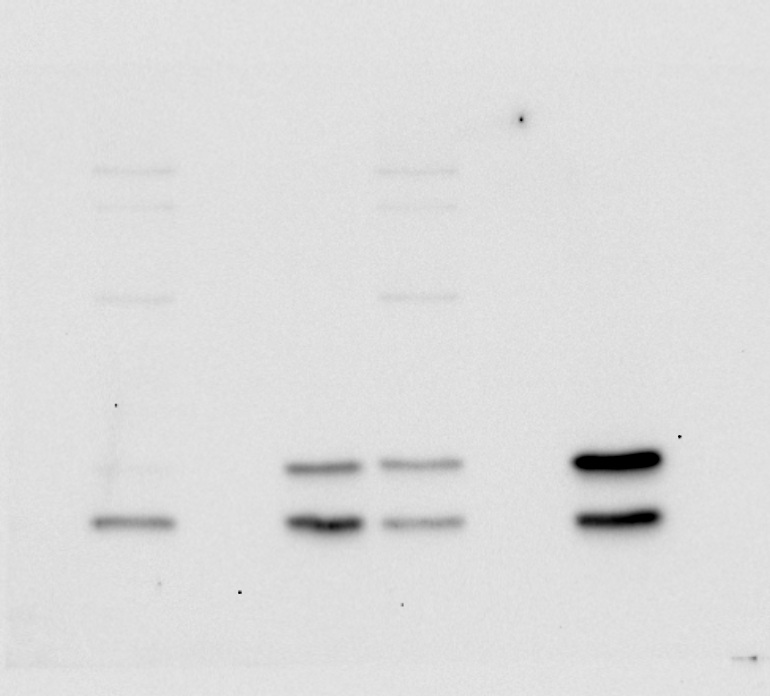

Supplement: Figure 1—source data 2. [file elife-87714-fig1-data2.zip › Figure 1-Source Data 2/Figure 1C Western blots/WB H3K9acK14ac.tif]

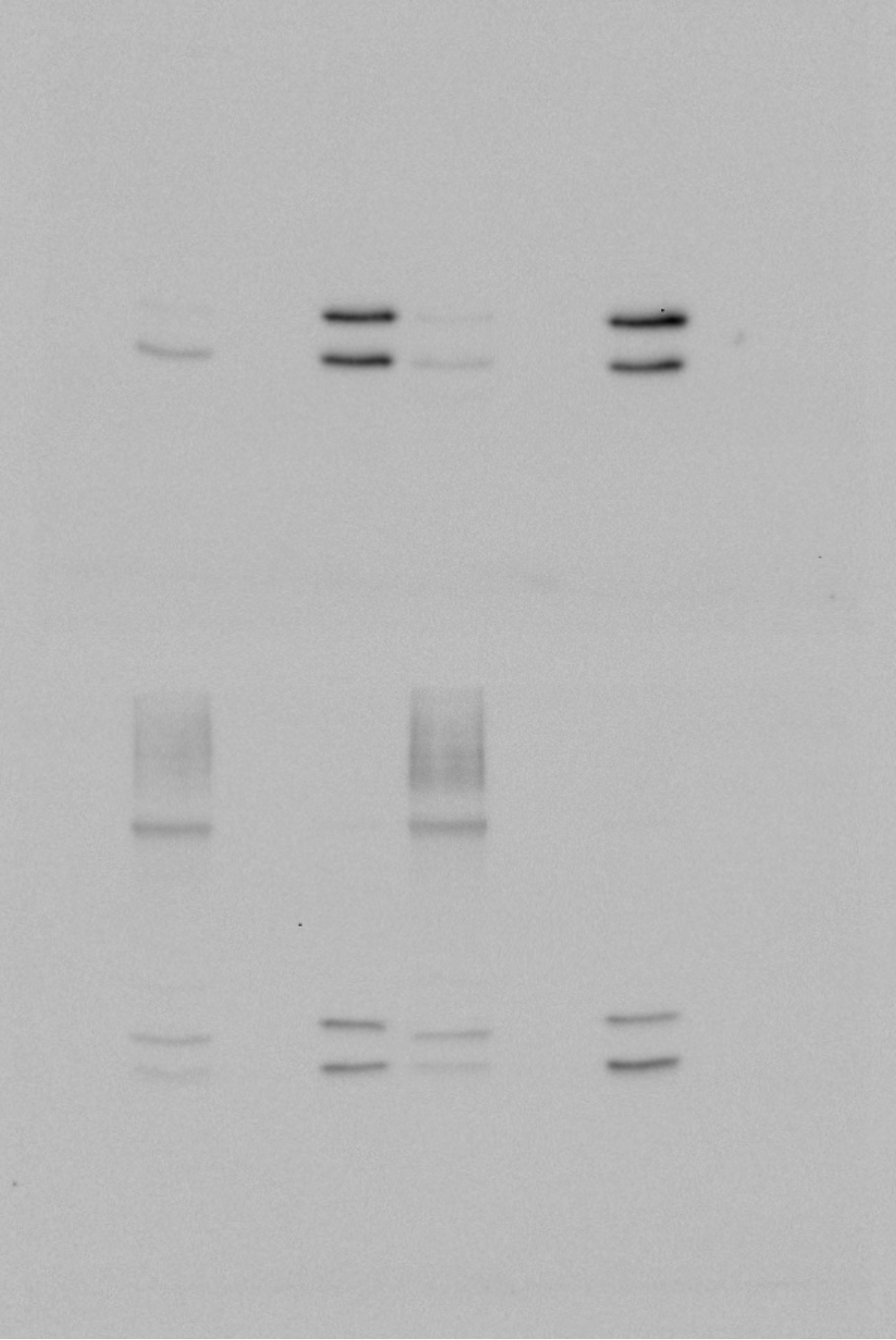

Supplement: Figure 1—source data 2. [file elife-87714-fig1-data2.zip › Figure 1-Source Data 2/Figure 1C Western blots/WB H3K9me1_H3K27me3.tif]

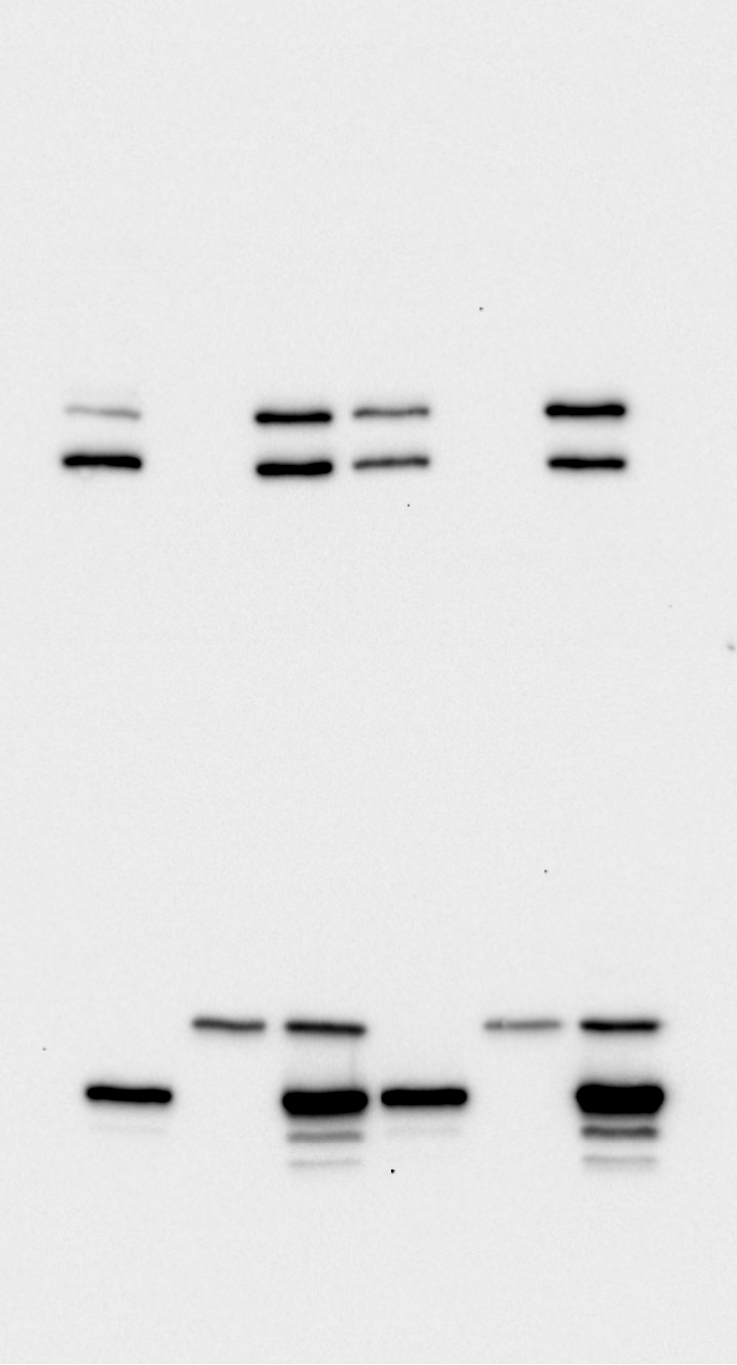

Supplement: Figure 1—source data 2. [file elife-87714-fig1-data2.zip › Figure 1-Source Data 2/Figure 1C Western blots/WB H3_HA.tif]

**Figure 1C**

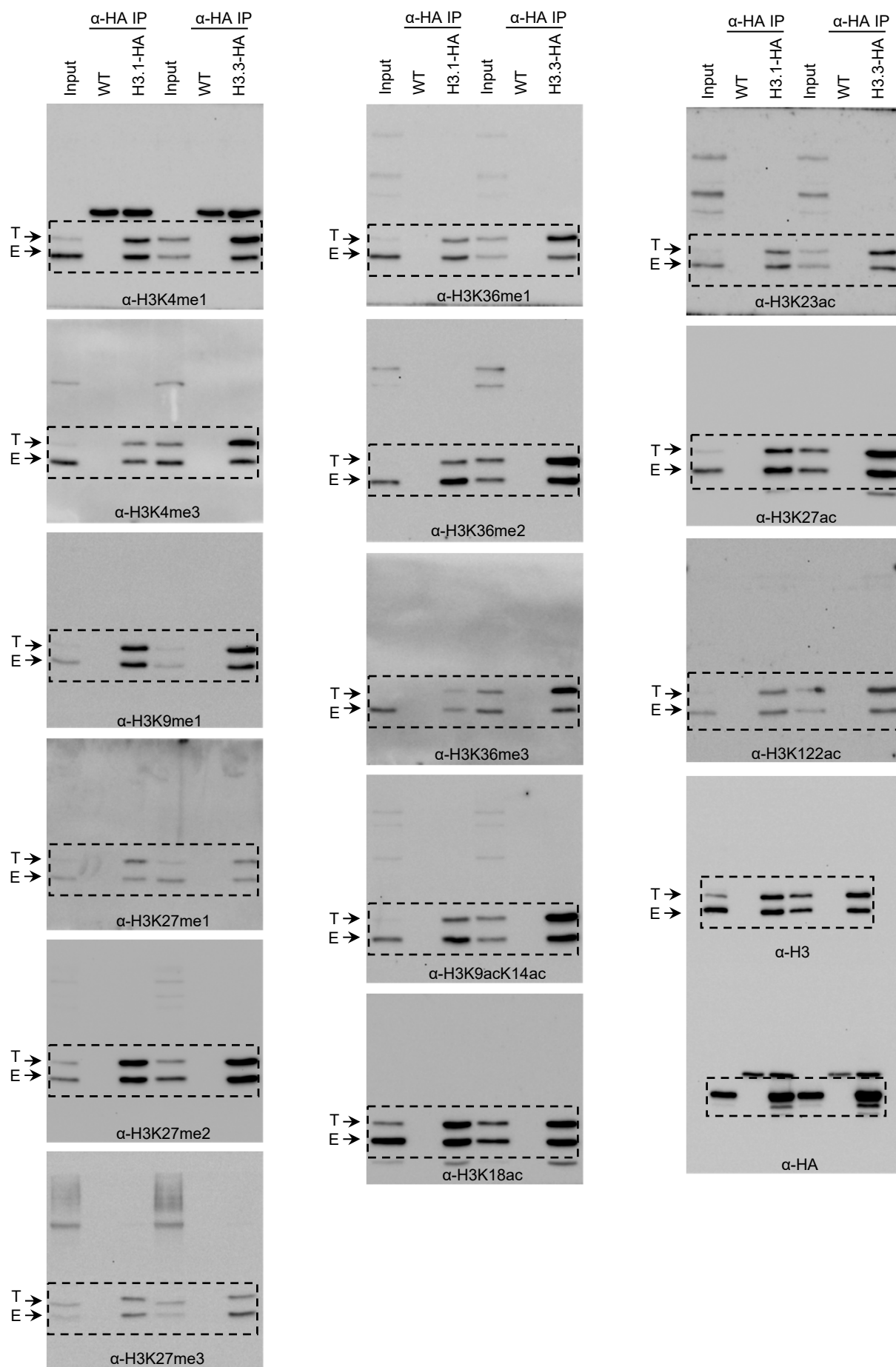

Supplement: Figure 1—source data 2. [file elife-87714-fig1-data2.zip › Figure 1-Source Data 2/WB data Figure 1C.pdf]

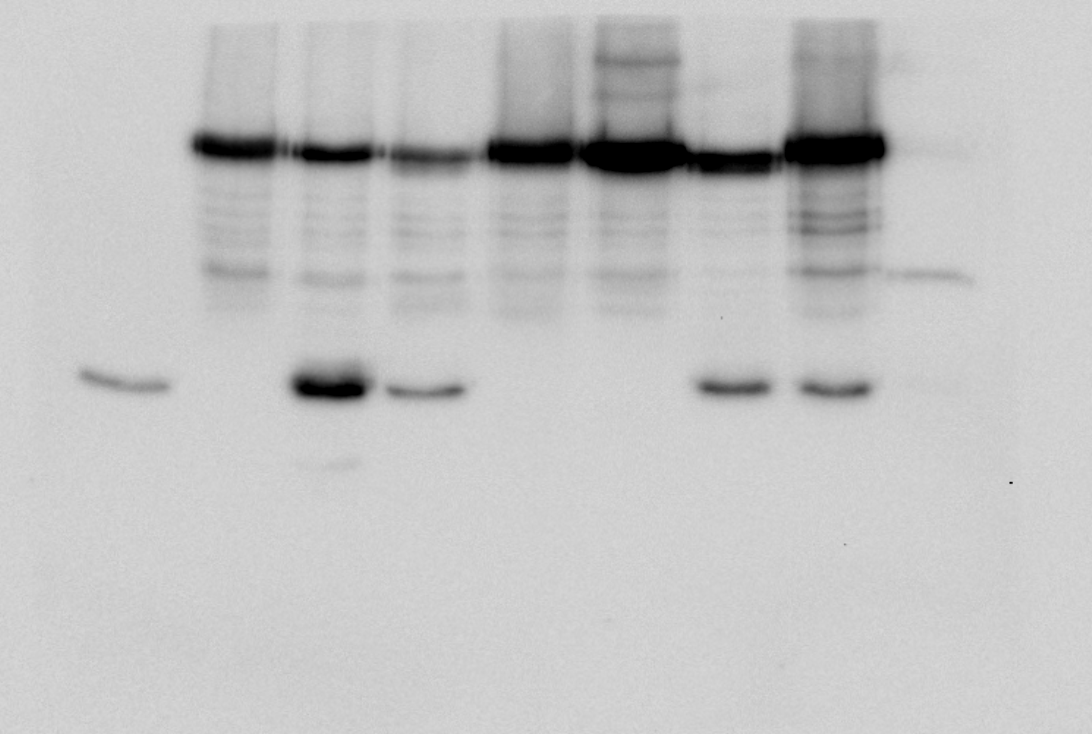

Supplement: Figure 1—source data 3. [file elife-87714-fig1-data3.zip › Figure 1-Source Data 3/Figure 1F_left panels Western blots/WB H2A.W.6.tif]

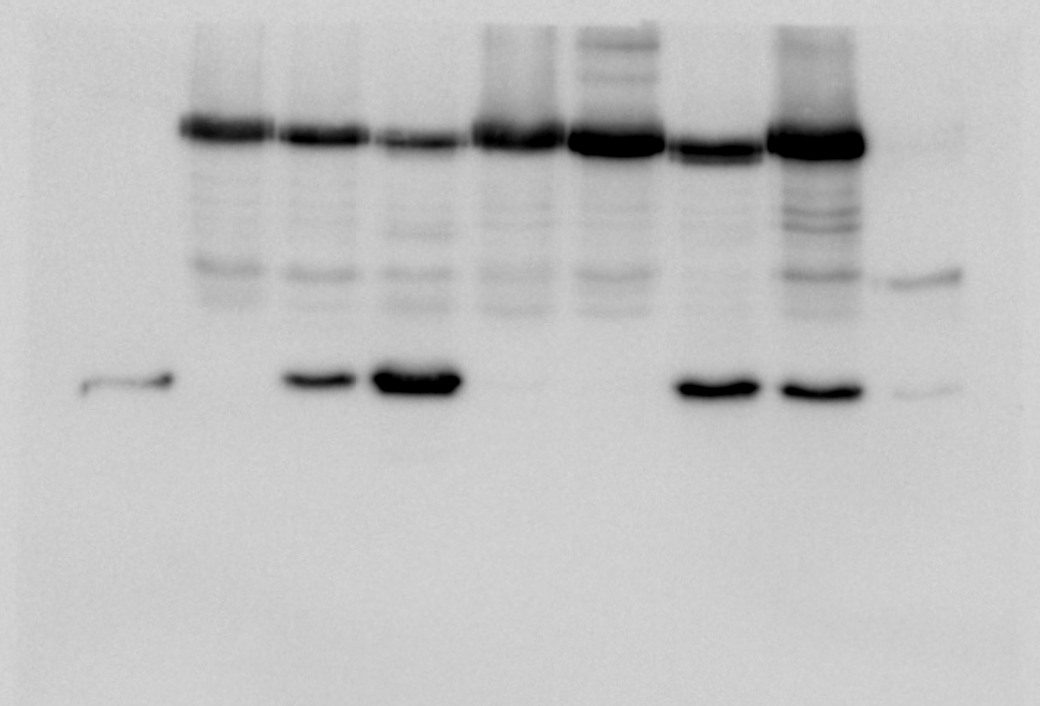

Supplement: Figure 1—source data 3. [file elife-87714-fig1-data3.zip › Figure 1-Source Data 3/Figure 1F_left panels Western blots/WB H2A.W.7.tif]

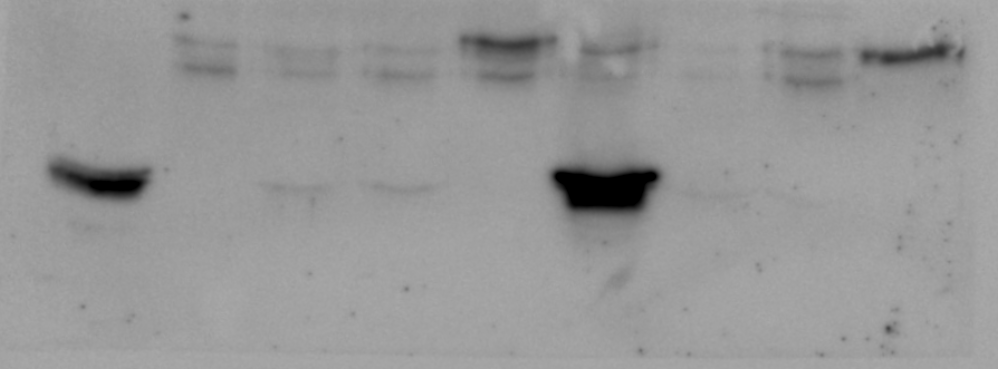

Supplement: Figure 1—source data 3. [file elife-87714-fig1-data3.zip › Figure 1-Source Data 3/Figure 1F_left panels Western blots/WB H2A.X.tif]

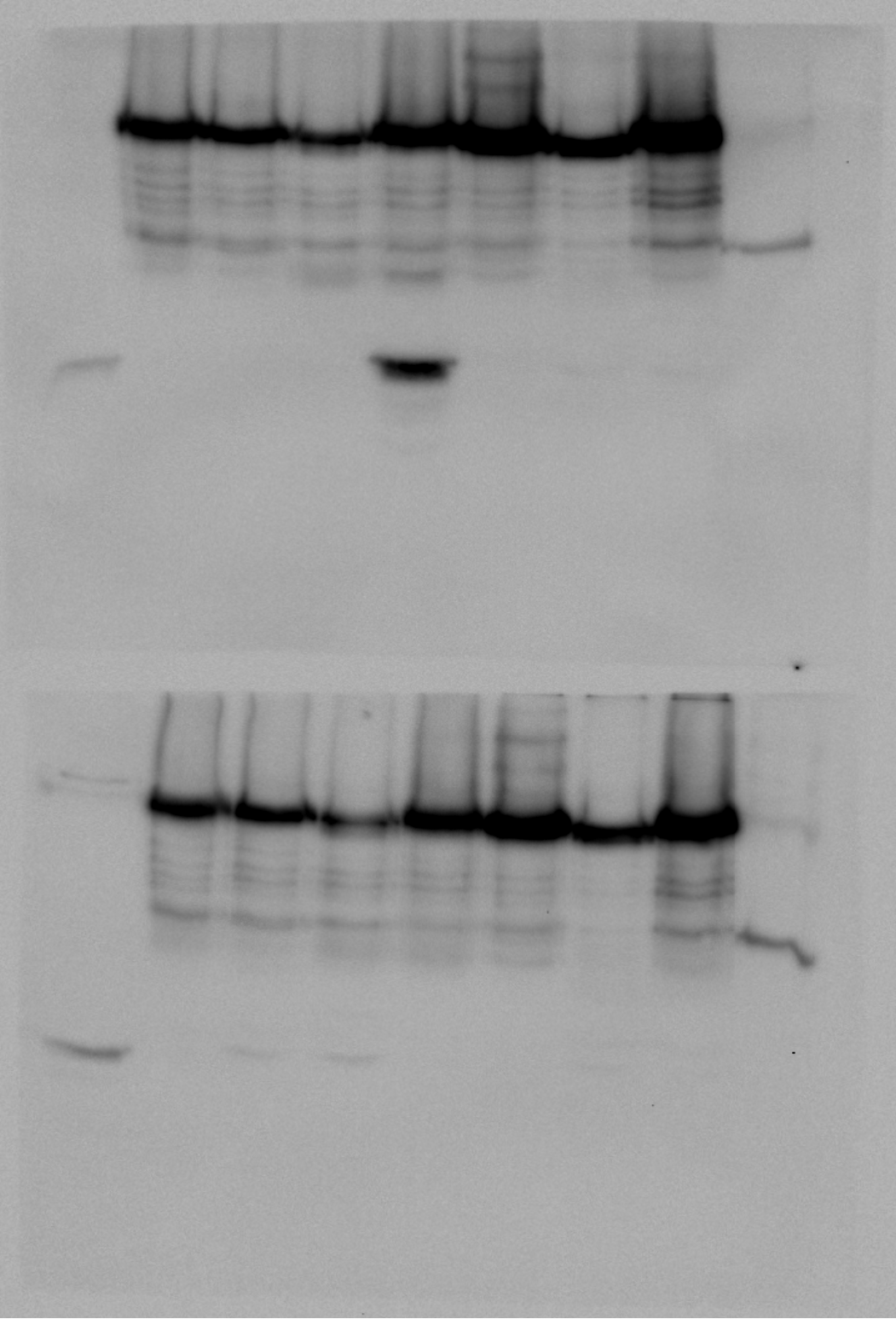

Supplement: Figure 1—source data 3. [file elife-87714-fig1-data3.zip › Figure 1-Source Data 3/Figure 1F_left panels Western blots/WB H2A.Z.9_H2A.13.tif]

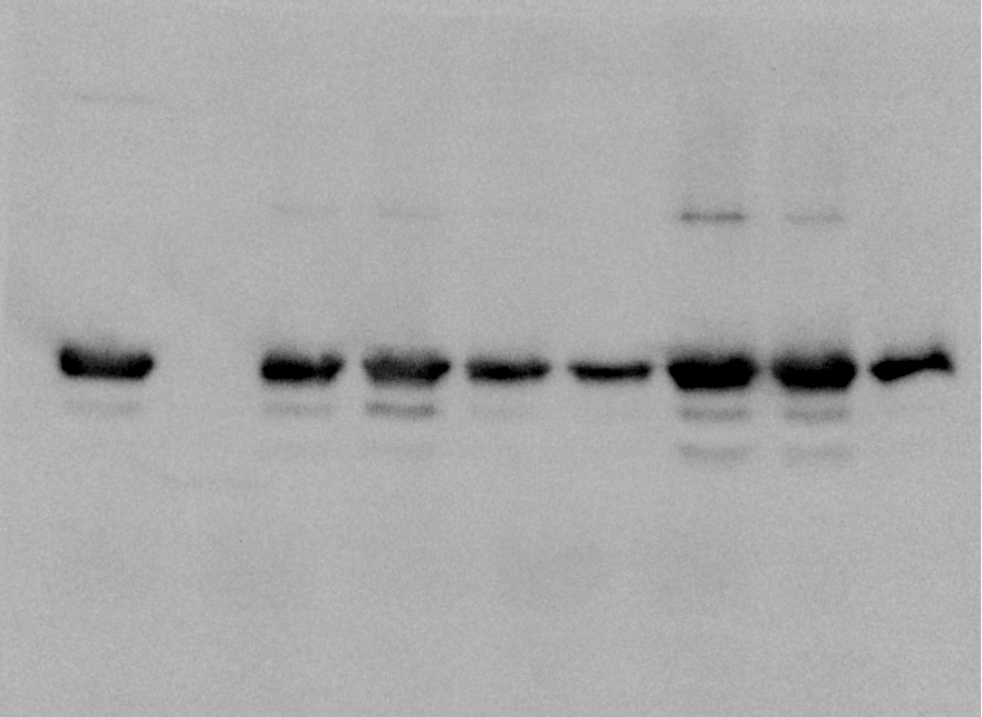

Supplement: Figure 1—source data 3. [file elife-87714-fig1-data3.zip › Figure 1-Source Data 3/Figure 1F_left panels Western blots/WB H3.tif]

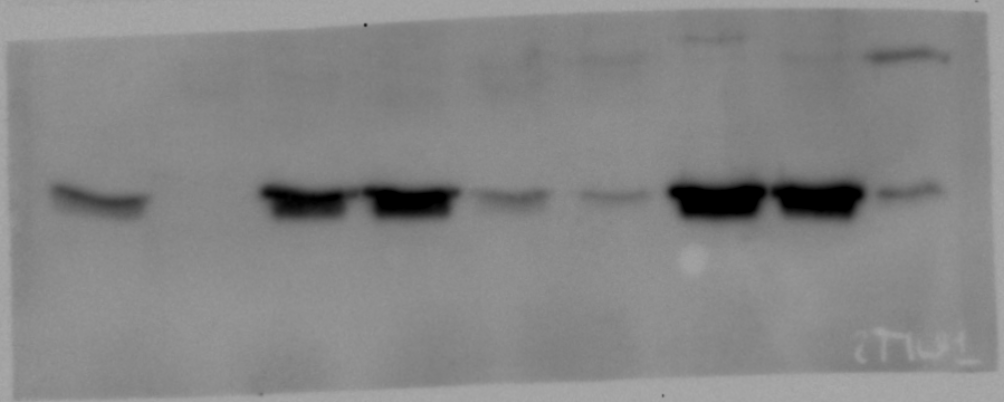

Supplement: Figure 1—source data 3. [file elife-87714-fig1-data3.zip › Figure 1-Source Data 3/Figure 1F_left panels Western blots/WB H3K27me1.tif]

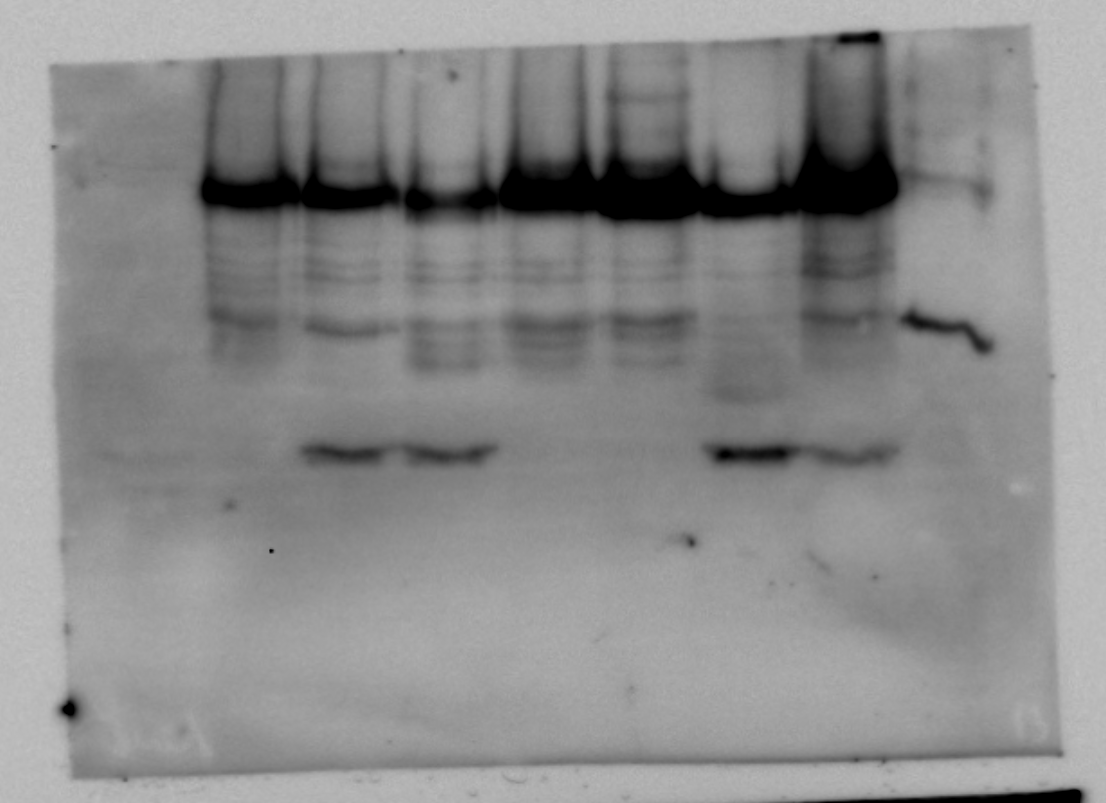

Supplement: Figure 1—source data 3. [file elife-87714-fig1-data3.zip › Figure 1-Source Data 3/Figure 1F_left panels Western blots/WB H3K9me1.tif]

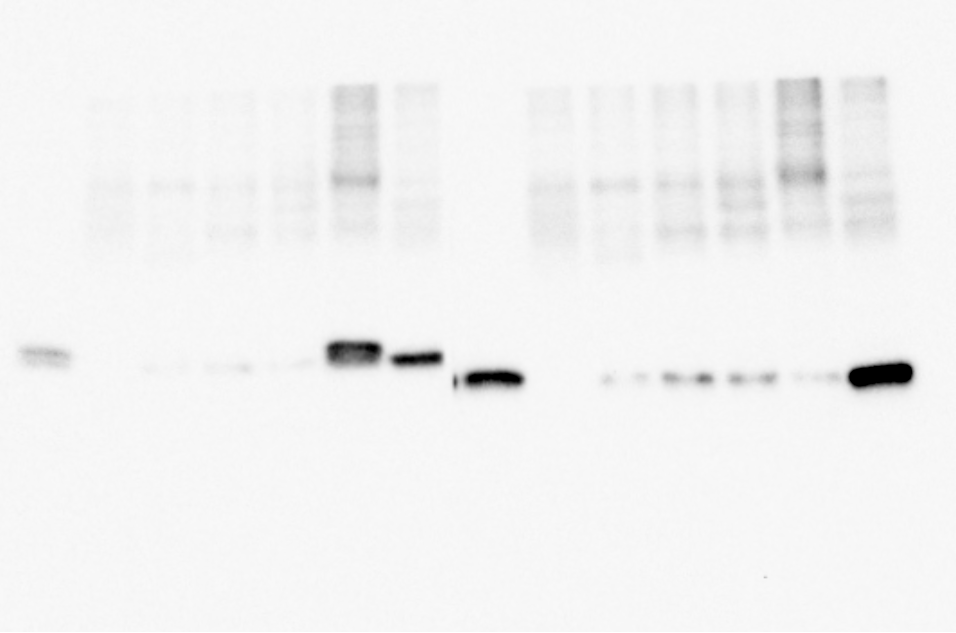

Supplement: Figure 1—source data 3. [file elife-87714-fig1-data3.zip › Figure 1-Source Data 3/Figure 1F_midle panels Western blots/WB H2A.X_H2A.13.tif]

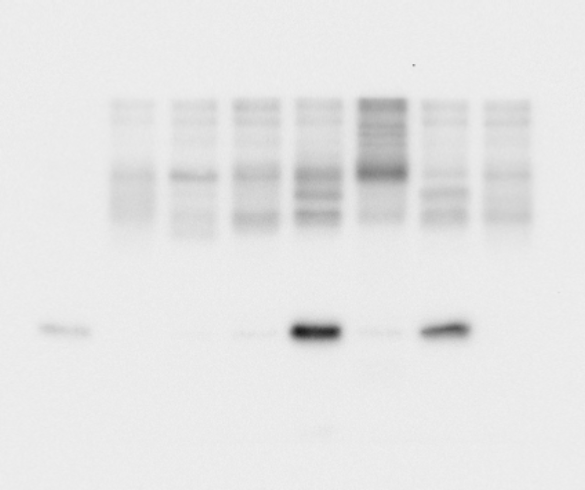

Supplement: Figure 1—source data 3. [file elife-87714-fig1-data3.zip › Figure 1-Source Data 3/Figure 1F_midle panels Western blots/WB H2A.Z.9.tif]

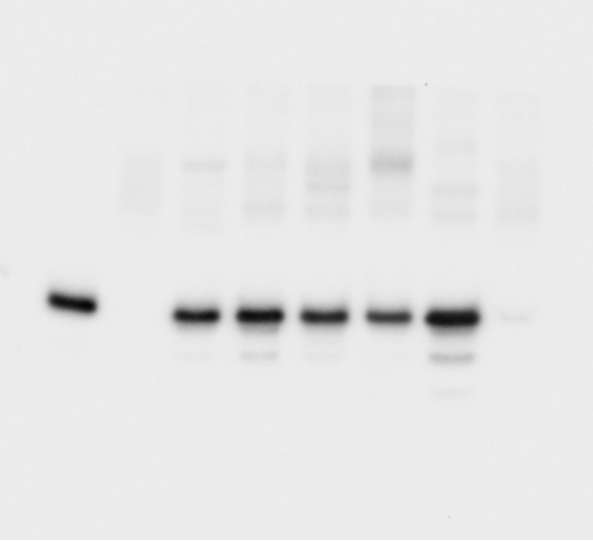

Supplement: Figure 1—source data 3. [file elife-87714-fig1-data3.zip › Figure 1-Source Data 3/Figure 1F_midle panels Western blots/WB H3.tif]

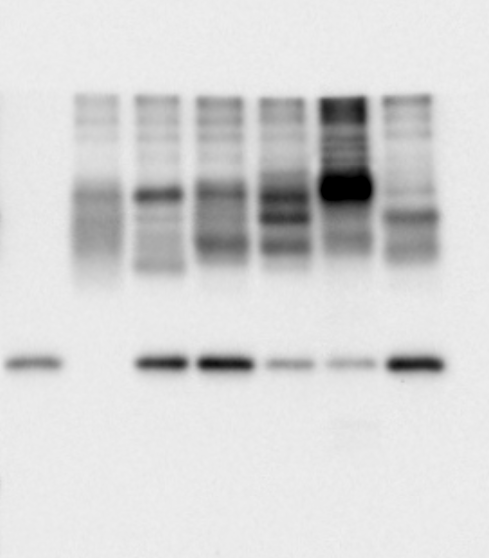

Supplement: Figure 1—source data 3. [file elife-87714-fig1-data3.zip › Figure 1-Source Data 3/Figure 1F_midle panels Western blots/WB H3K27me1.tif]

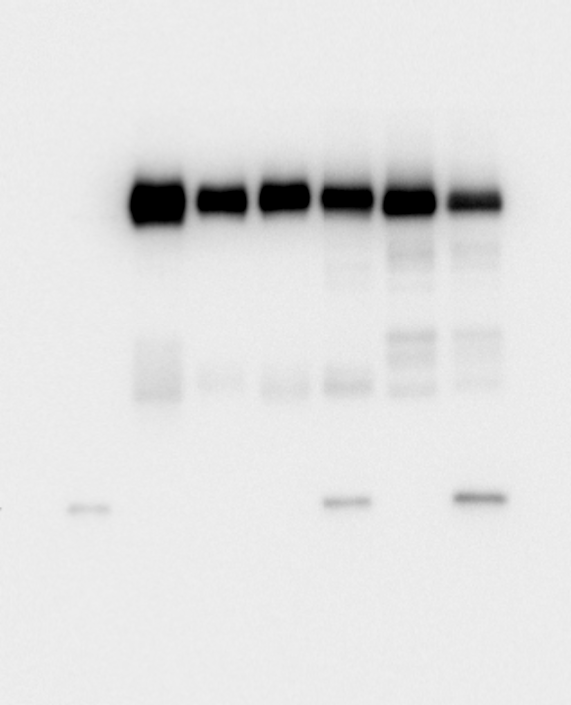

Supplement: Figure 1—source data 3. [file elife-87714-fig1-data3.zip › Figure 1-Source Data 3/Figure 1F_midle panels Western blots/WB H3K27me3.tif]

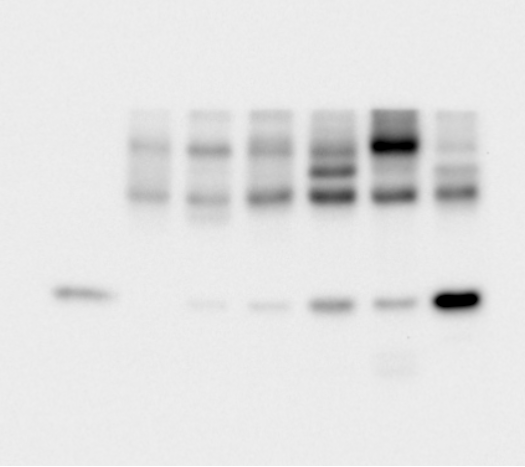

Supplement: Figure 1—source data 3. [file elife-87714-fig1-data3.zip › Figure 1-Source Data 3/Figure 1F_midle panels Western blots/WB H3K36me3.tif]

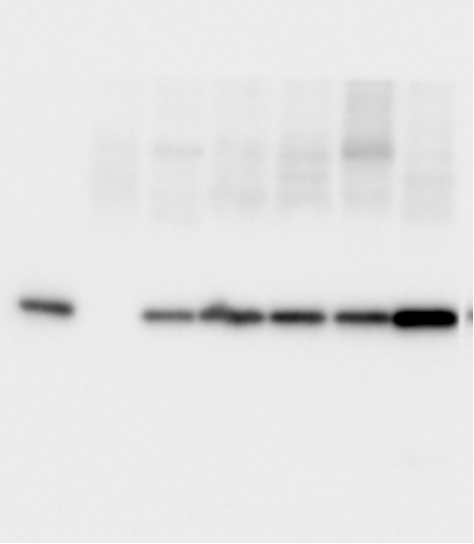

Supplement: Figure 1—source data 3. [file elife-87714-fig1-data3.zip › Figure 1-Source Data 3/Figure 1F_midle panels Western blots/WB H3K4me1.tif]

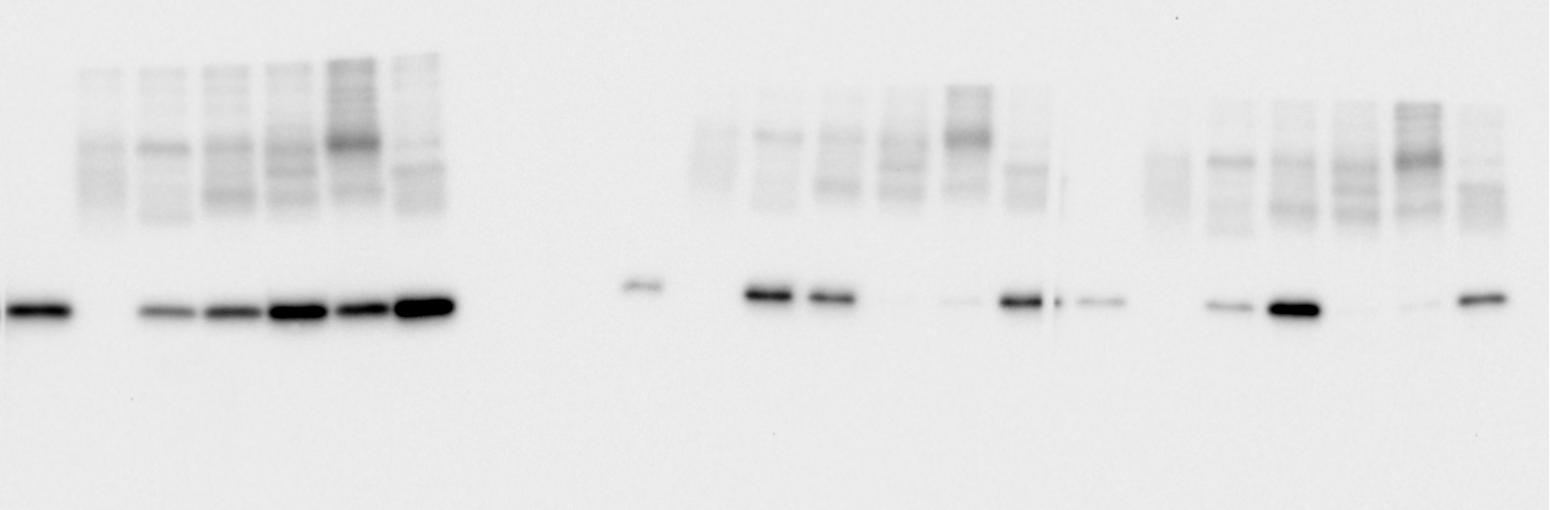

Supplement: Figure 1—source data 3. [file elife-87714-fig1-data3.zip › Figure 1-Source Data 3/Figure 1F_midle panels Western blots/WB H3K4me3_H2A.W.7.tif]

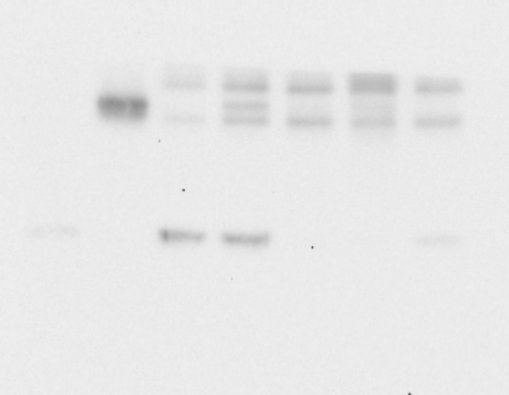

Supplement: Figure 1—source data 3. [file elife-87714-fig1-data3.zip › Figure 1-Source Data 3/Figure 1F_midle panels Western blots/WB H3K9me2.tif]

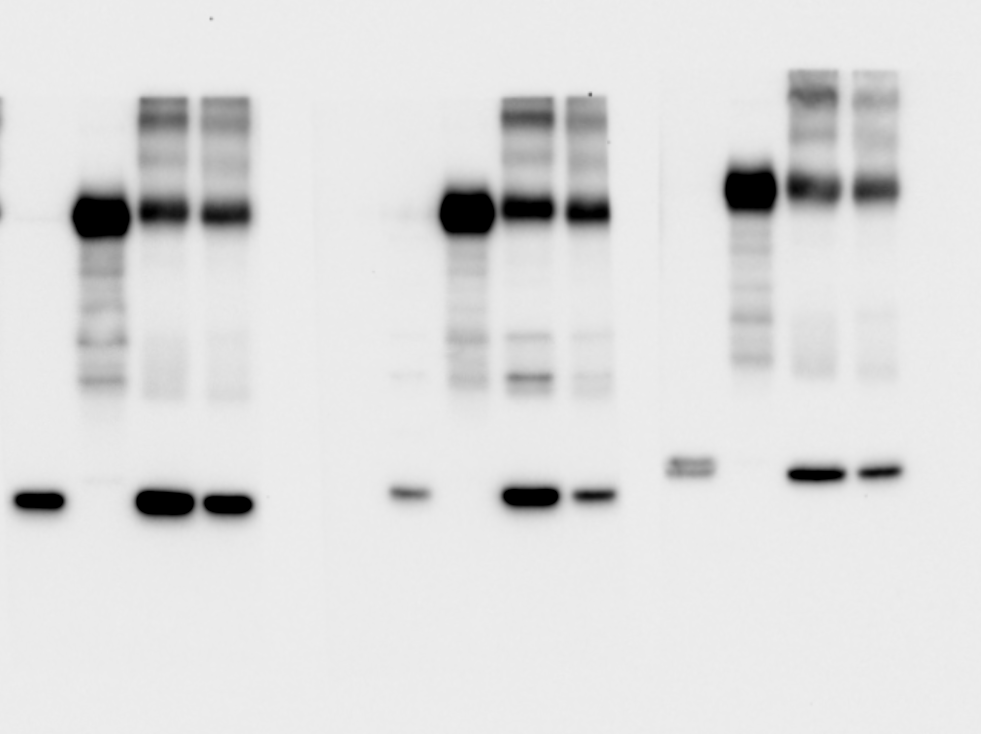

Supplement: Figure 1—source data 3. [file elife-87714-fig1-data3.zip › Figure 1-Source Data 3/Figure 1F_right panels Western blots/WB H2A_H2A.Z.9_H2A.X.tif]

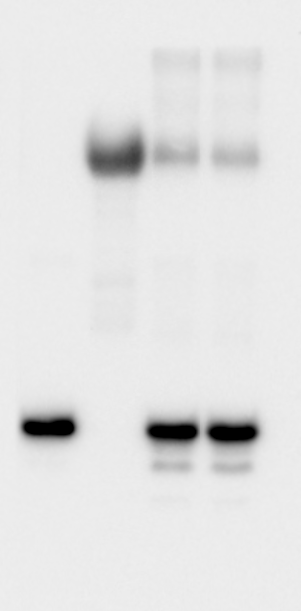

Supplement: Figure 1—source data 3. [file elife-87714-fig1-data3.zip › Figure 1-Source Data 3/Figure 1F_right panels Western blots/WB H3.tif]

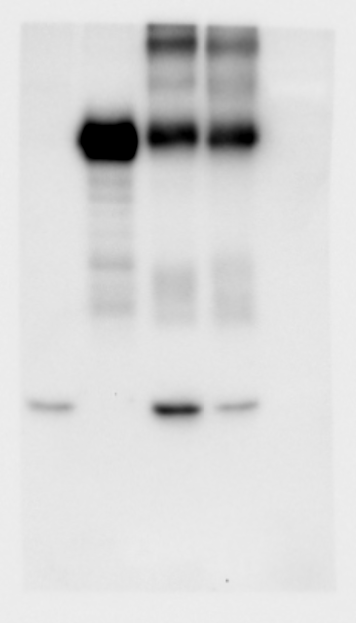

Supplement: Figure 1—source data 3. [file elife-87714-fig1-data3.zip › Figure 1-Source Data 3/Figure 1F_right panels Western blots/WB H3K27me1.tif]

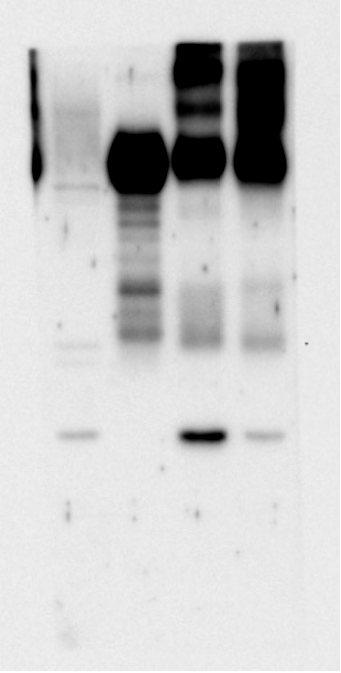

Supplement: Figure 1—source data 3. [file elife-87714-fig1-data3.zip › Figure 1-Source Data 3/Figure 1F_right panels Western blots/WB H3K27me3.tif]

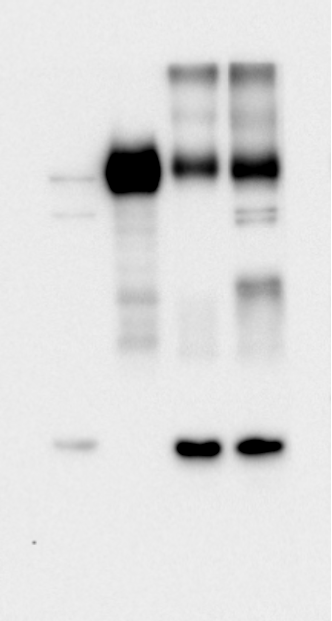

Supplement: Figure 1—source data 3. [file elife-87714-fig1-data3.zip › Figure 1-Source Data 3/Figure 1F_right panels Western blots/WB H3K36me3.tif]

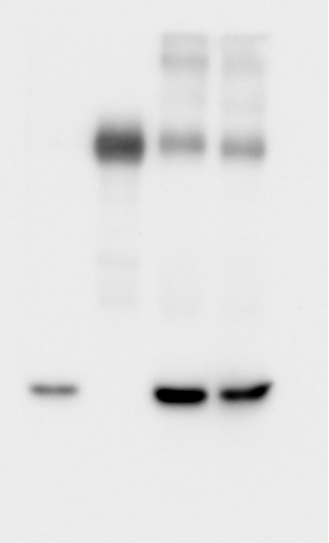

Supplement: Figure 1—source data 3. [file elife-87714-fig1-data3.zip › Figure 1-Source Data 3/Figure 1F_right panels Western blots/WB H3K4me3.tif]

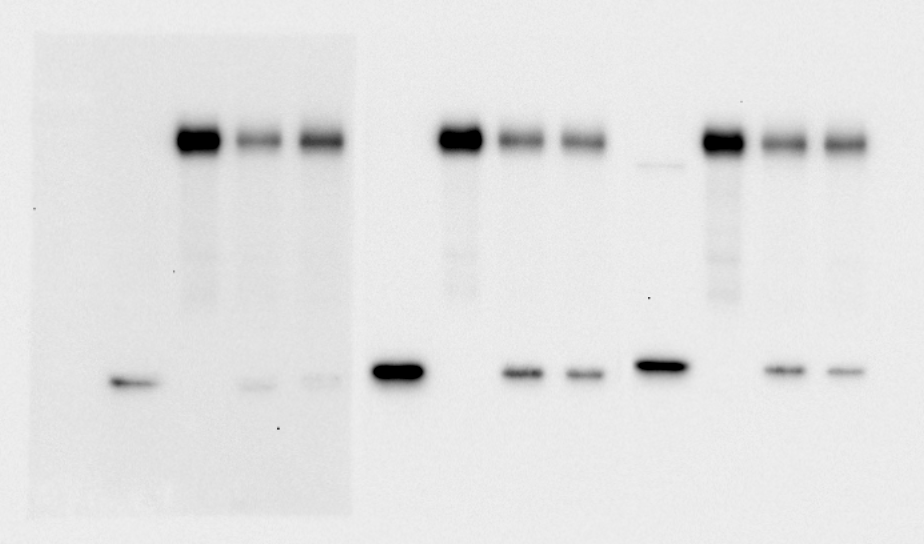

Supplement: Figure 1—source data 3. [file elife-87714-fig1-data3.zip › Figure 1-Source Data 3/Figure 1F_right panels Western blots/WB H3K9me1_H2A.W.6_H2A.W.7.tif]

Figure 1F – left panels

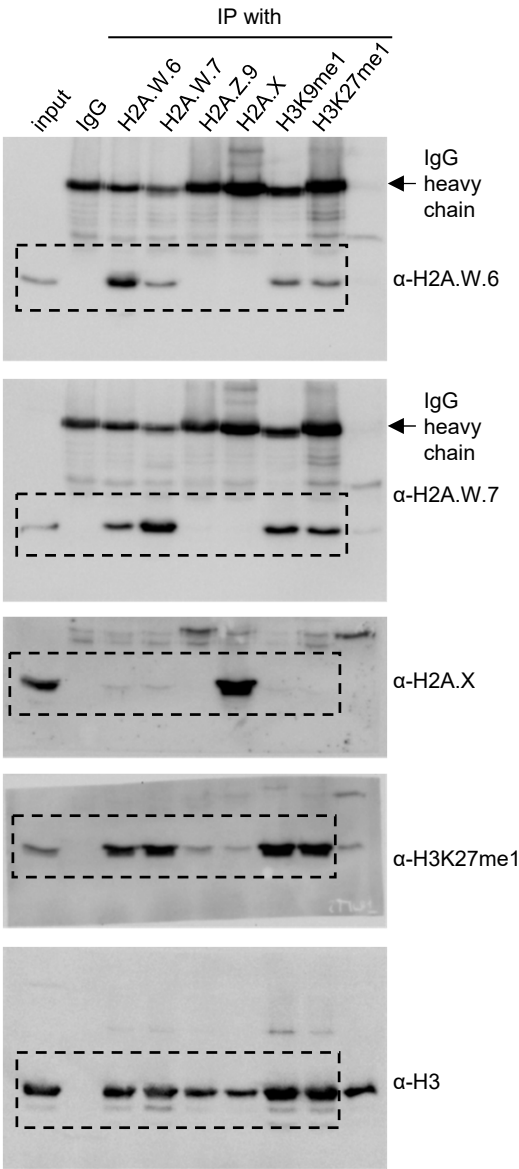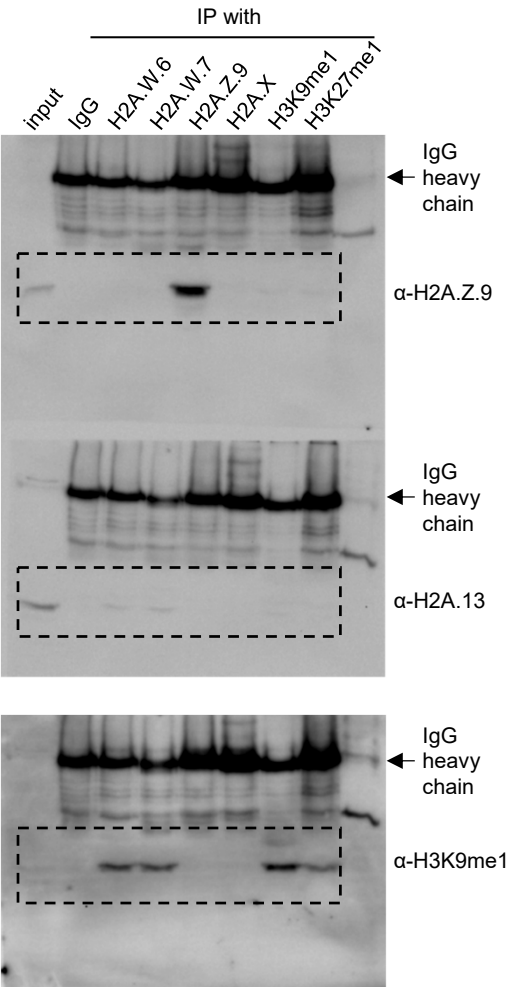

**Figure 1F – middle panels**

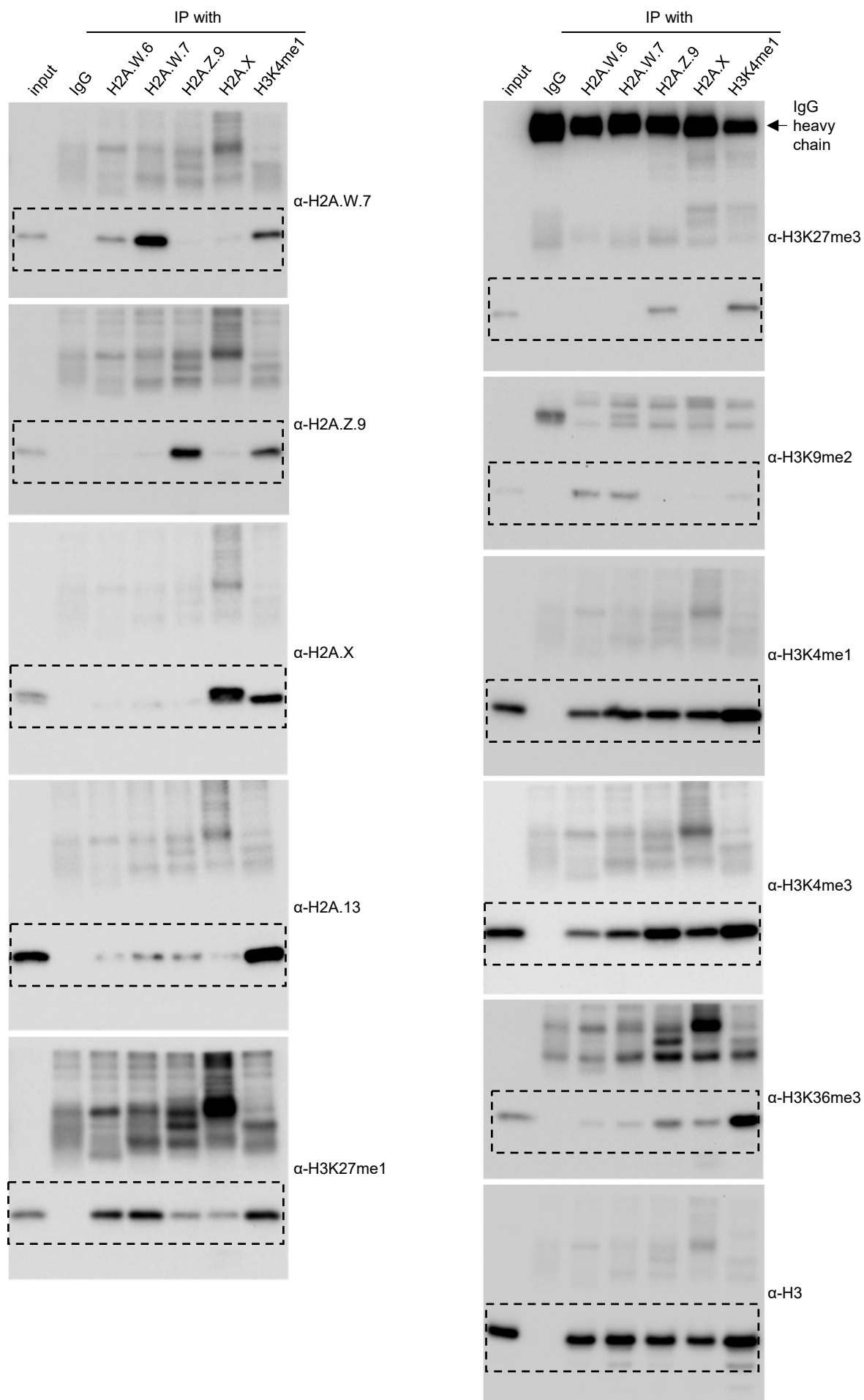

Figure 1F – right panels

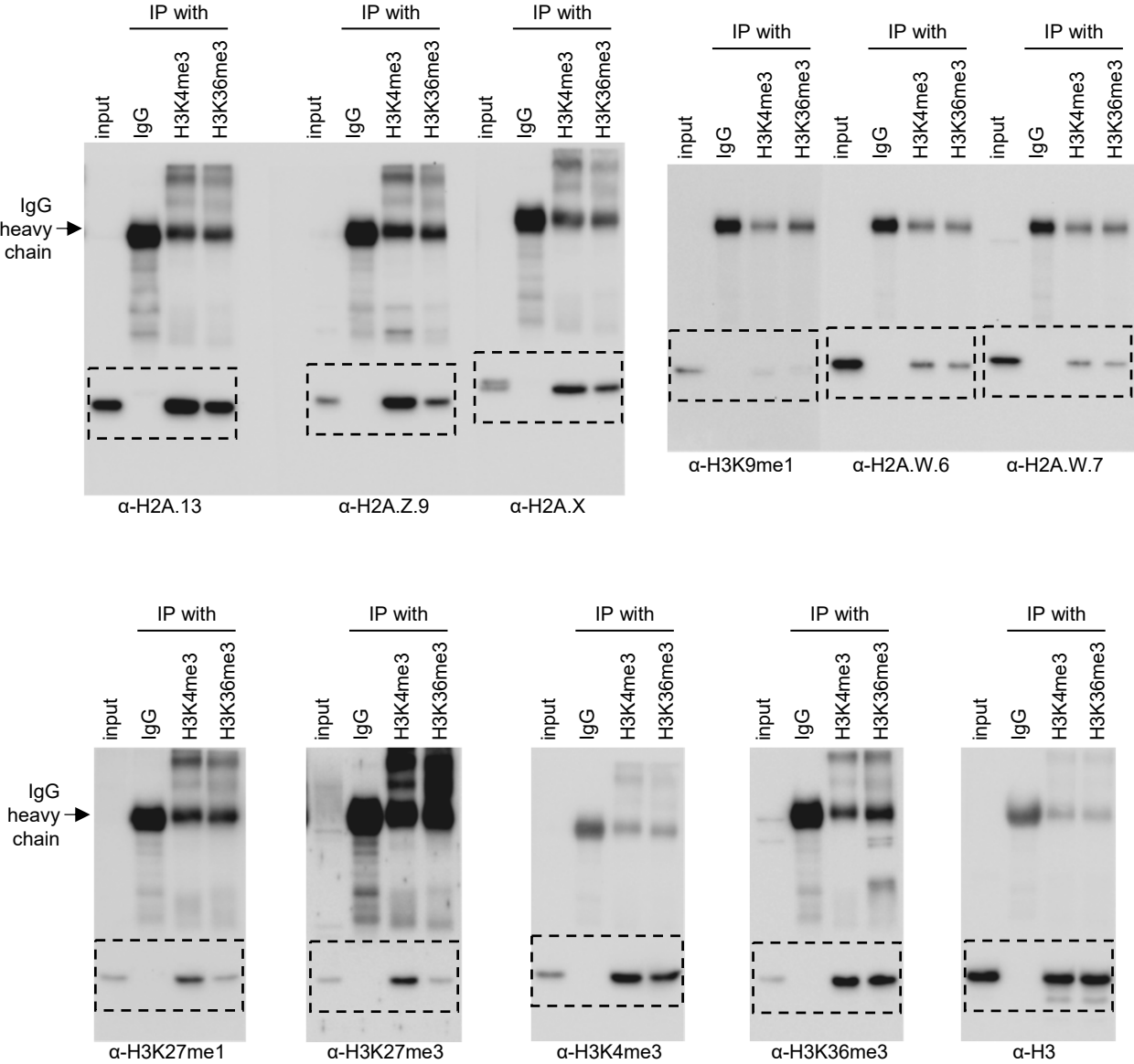

Supplement: Figure 1—source data 3. [file elife-87714-fig1-data3.zip › Figure 1-Source Data 3/WB data Figure 1F.pdf]

Figure 1-Figure Supplement 1B

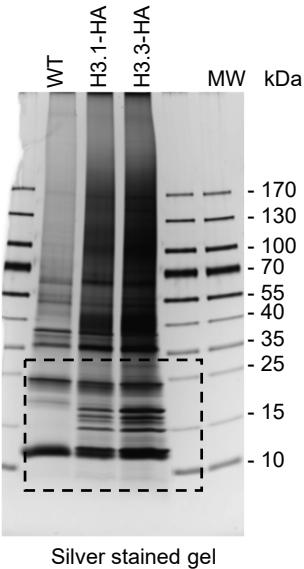

Supplement: Figure 1—figure supplement 1—source data 1. [file elife-87714-fig1-figsupp1-data1.zip › Figure 1–Figure Supplement 1–Source Data 1/Figure 1-Figure Supplement 1B.pdf]

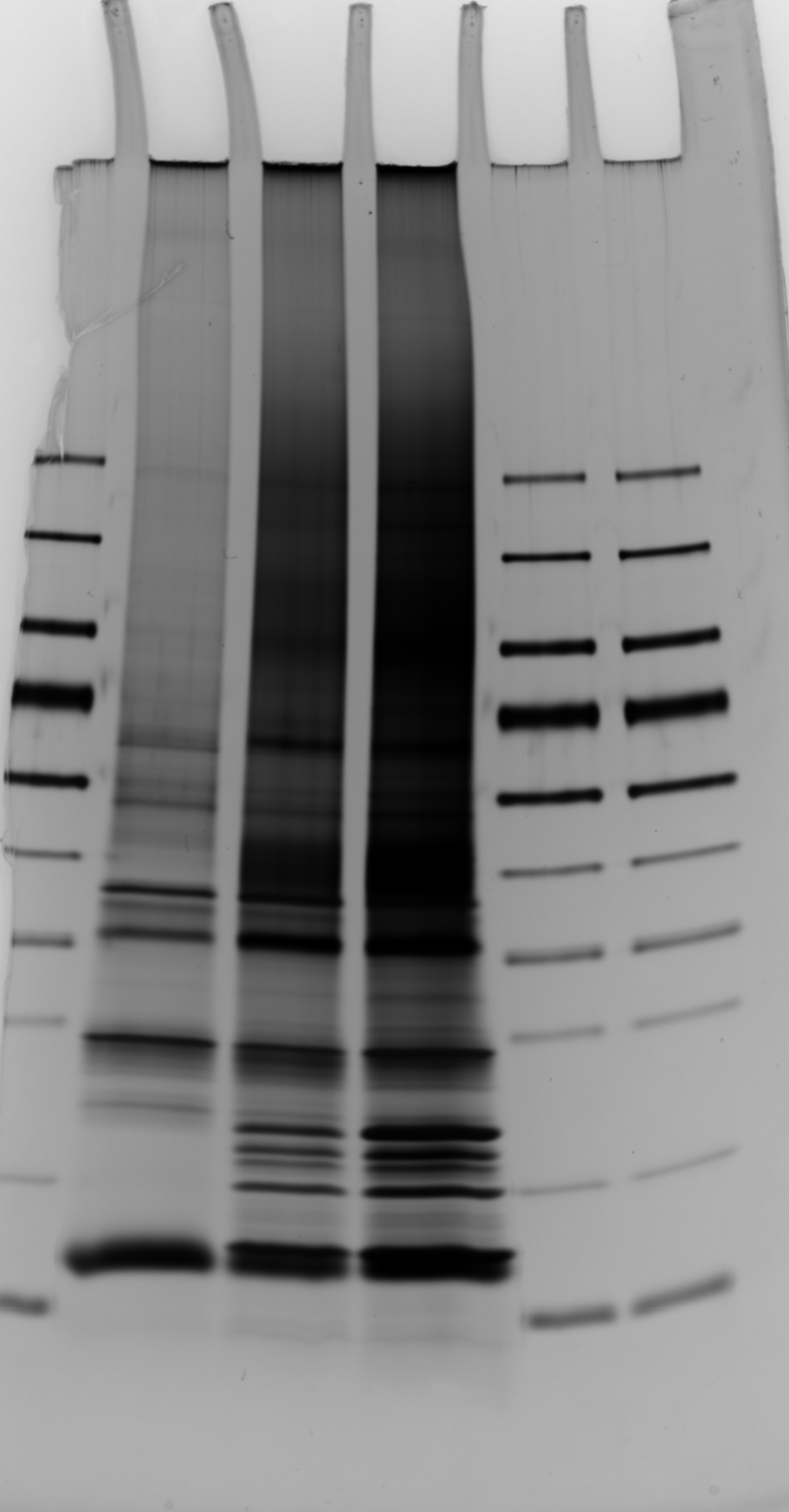

Supplement: Figure 1—figure supplement 1—source data 1. [file elife-87714-fig1-figsupp1-data1.zip › Figure 1–Figure Supplement 1–Source Data 1/Figure1-Figure supplement 1B/H3.1_H3.3_IPs_silverstained gel.tif]

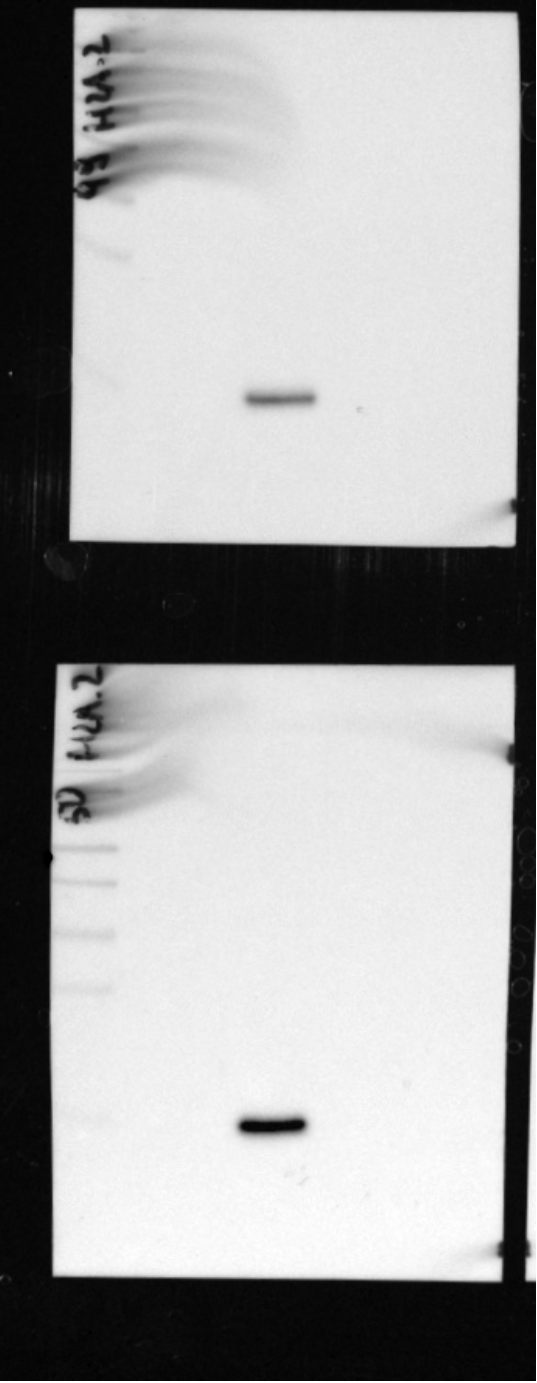

Supplement: Figure 2—figure supplement 1—source data 1. [file elife-87714-fig2-figsupp1-data1.zip › Figure 2–Figure Supplement 1–Source Data 1/Figure 2-Figure Supplement 1B/H2A.2_antibody test.tif]

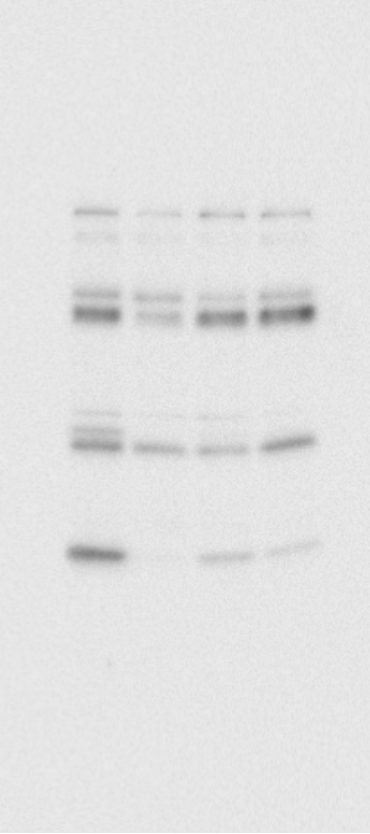

Supplement: Figure 2—figure supplement 1—source data 1. [file elife-87714-fig2-figsupp1-data1.zip › Figure 2–Figure Supplement 1–Source Data 1/Figure 2-Figure Supplement 1B/H2A.Z.11_antibody test.tif]

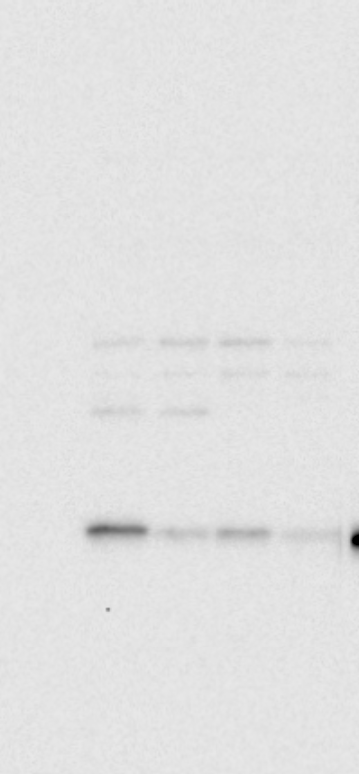

Supplement: Figure 2—figure supplement 1—source data 1. [file elife-87714-fig2-figsupp1-data1.zip › Figure 2–Figure Supplement 1–Source Data 1/Figure 2-Figure Supplement 1B/H2A.Z.9_antibody test.tif]

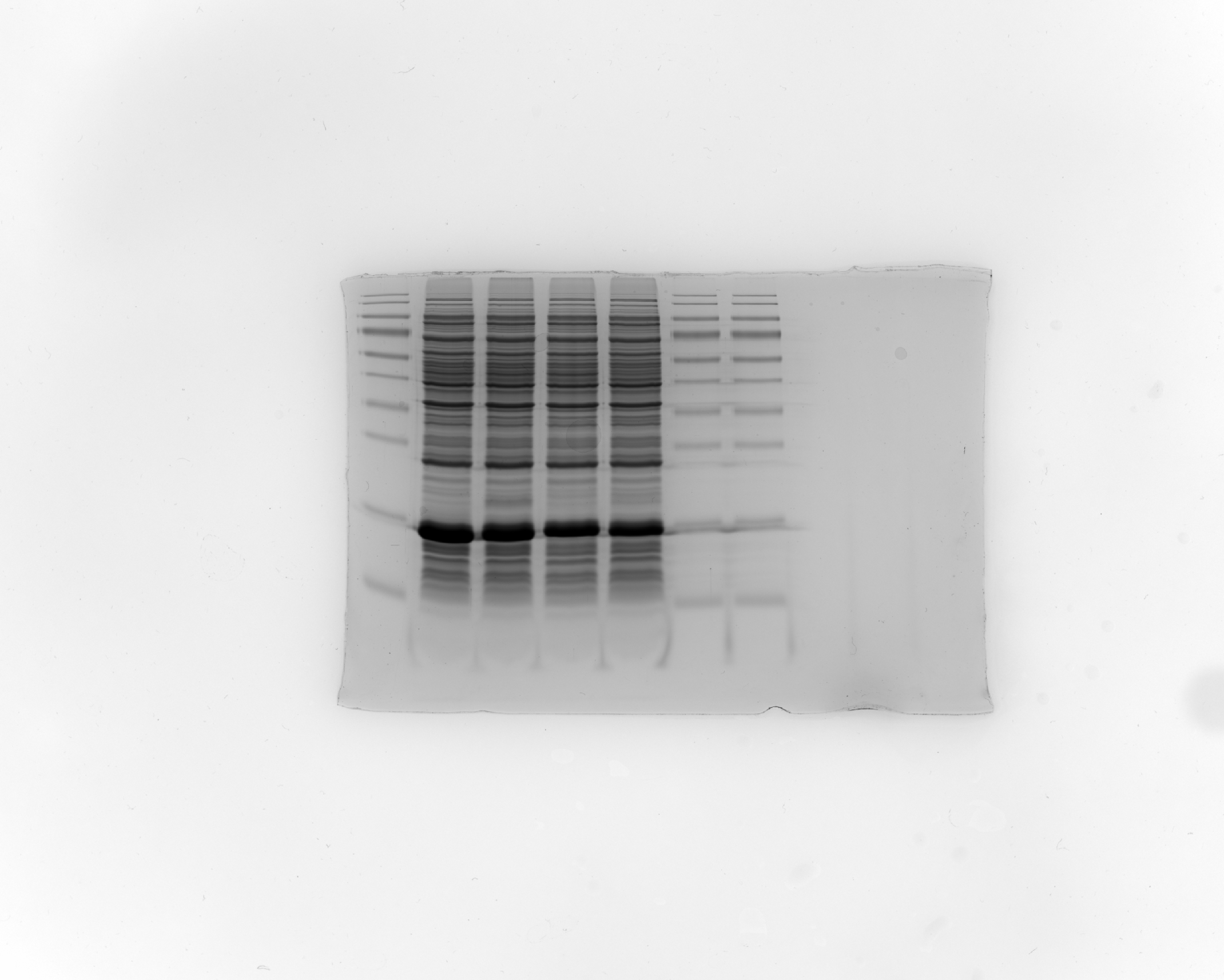

Supplement: Figure 2—figure supplement 1—source data 1. [file elife-87714-fig2-figsupp1-data1.zip › Figure 2–Figure Supplement 1–Source Data 1/Figure 2-Figure Supplement 1B/H2A_overexpression gel.tif]

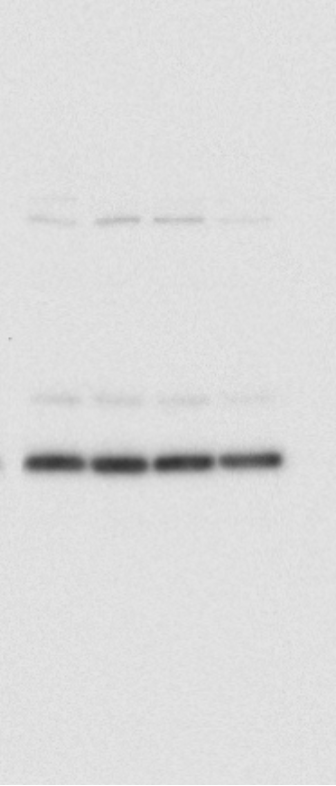

Supplement: Figure 2—figure supplement 1—source data 1. [file elife-87714-fig2-figsupp1-data1.zip › Figure 2–Figure Supplement 1–Source Data 1/Figure 2-Figure Supplement 1B/H3 loading control for H2A.Z antibody tests.tif]

Figure 2-Figure Supplement 1B

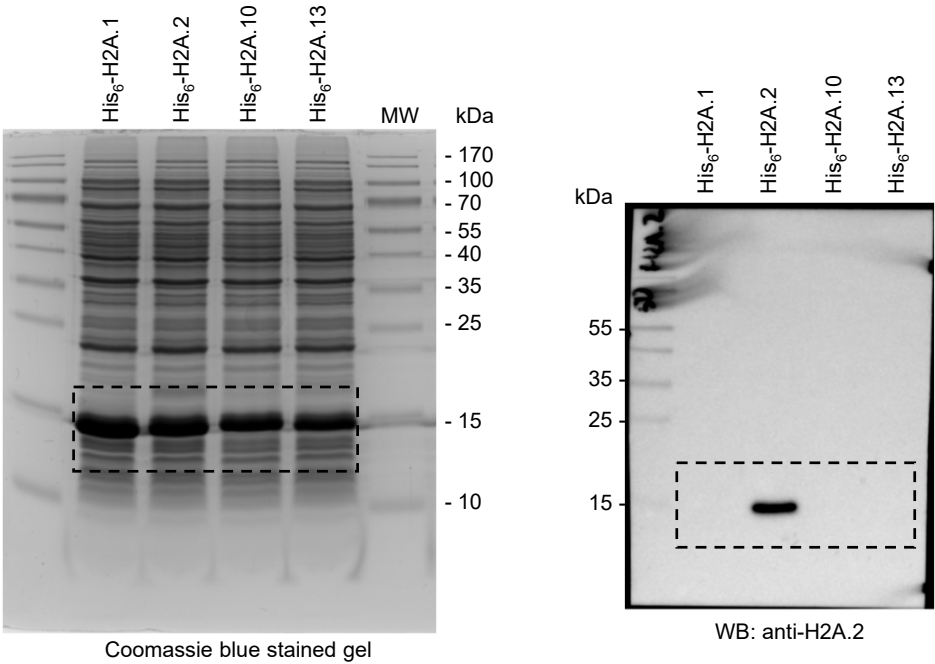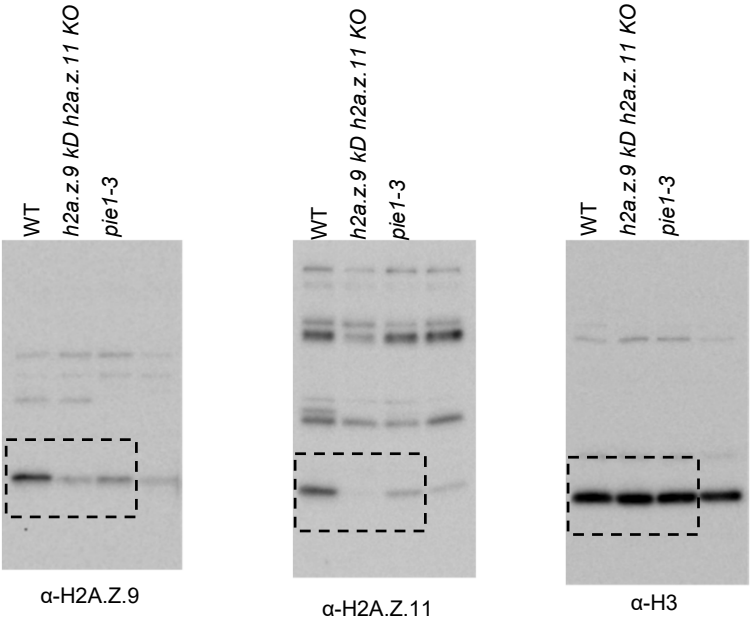

Supplement: Figure 2—figure supplement 1—source data 1. [file elife-87714-fig2-figsupp1-data1.zip › Figure 2–Figure Supplement 1–Source Data 1/Figure 2-Figure Supplement 1B.pdf]

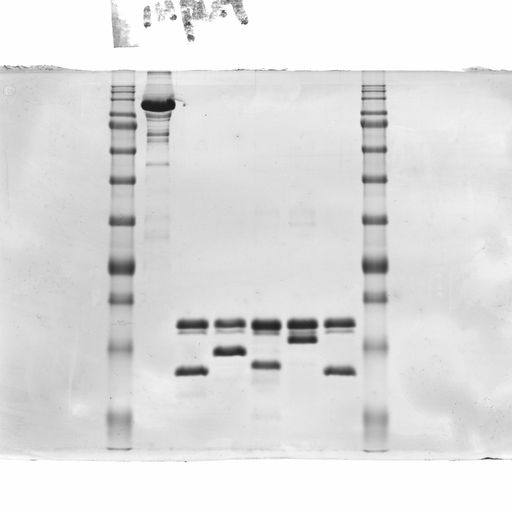

Supplement: Figure 3—source data 1. [file elife-87714-fig3-data1.zip › Figure 3-Source Data 1/Figure 3G protein gels/Input.tif]

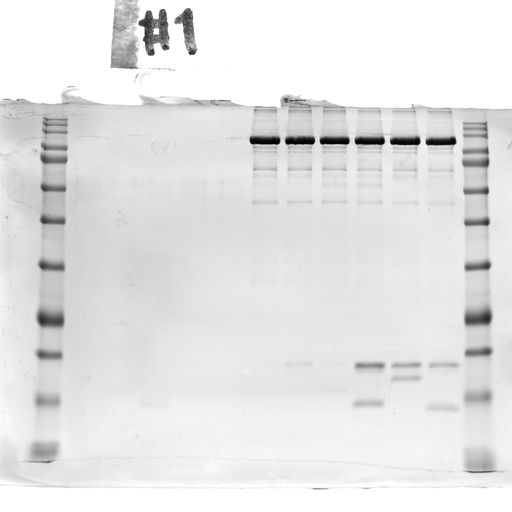

Supplement: Figure 3—source data 1. [file elife-87714-fig3-data1.zip › Figure 3-Source Data 1/Figure 3G protein gels/Pull-down.tif]

**Figure 3G**

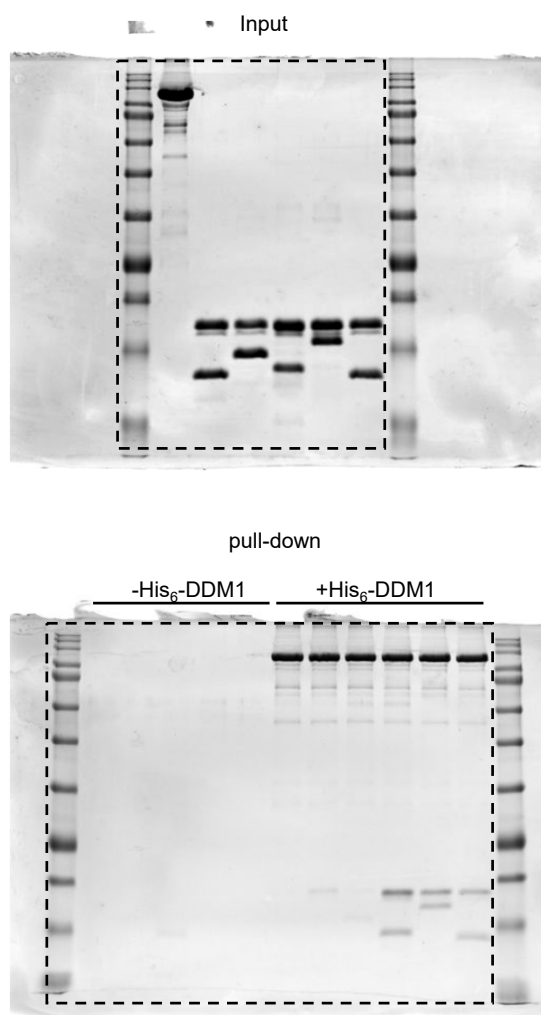

Supplement: Figure 3—source data 1. [file elife-87714-fig3-data1.zip › Figure 3-Source Data 1/Protein gels Figure 3G.pdf]

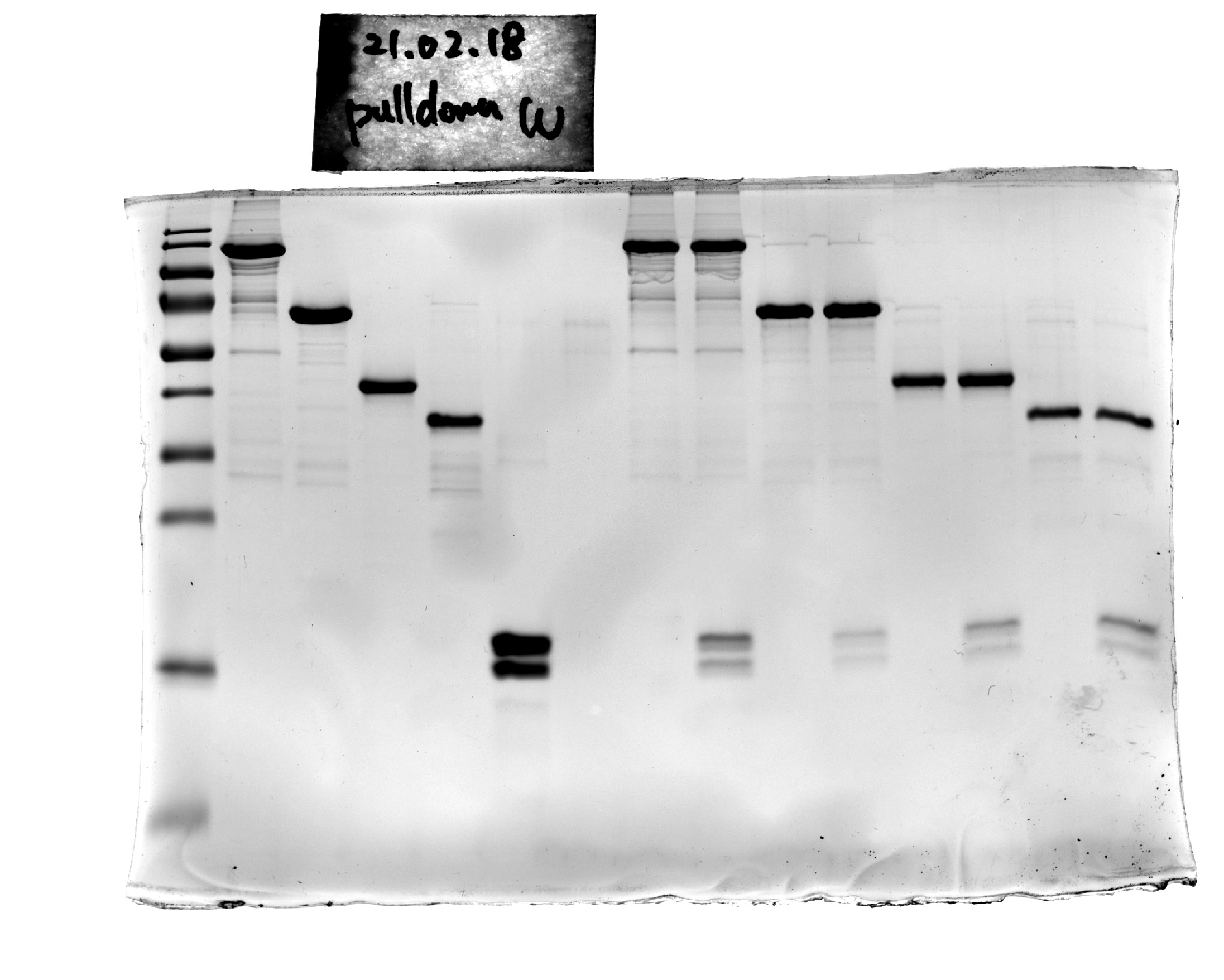

Supplement: Figure 3—figure supplement 2—source data 1. [file elife-87714-fig3-figsupp2-data1.zip › Figure 3-Figure Supplement 2-Source Data 1/Figure 3-Figure Supplement 2A/H2A.W-H2B.tif]

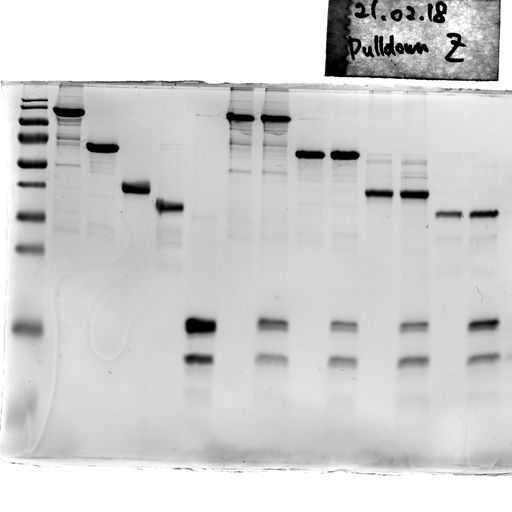

Supplement: Figure 3—figure supplement 2—source data 1. [file elife-87714-fig3-figsupp2-data1.zip › Figure 3-Figure Supplement 2-Source Data 1/Figure 3-Figure Supplement 2A/H2A.Z-H2B.tif]

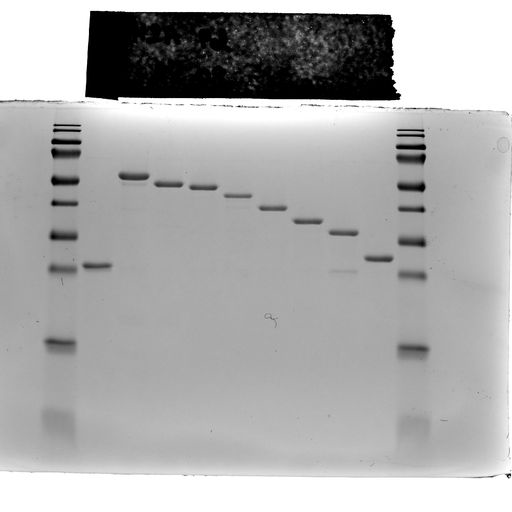

Supplement: Figure 3—figure supplement 2—source data 1. [file elife-87714-fig3-figsupp2-data1.zip › Figure 3-Figure Supplement 2-Source Data 1/Figure 3-Figure Supplement 2B/H2A-H2B pull-down.tif]

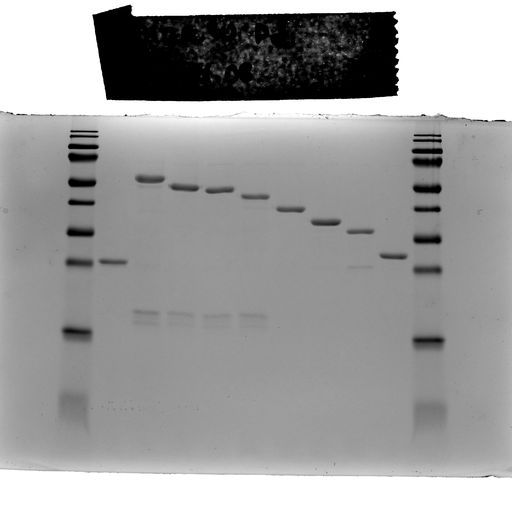

Supplement: Figure 3—figure supplement 2—source data 1. [file elife-87714-fig3-figsupp2-data1.zip › Figure 3-Figure Supplement 2-Source Data 1/Figure 3-Figure Supplement 2B/H2A.W-H2B pull-down.tif]

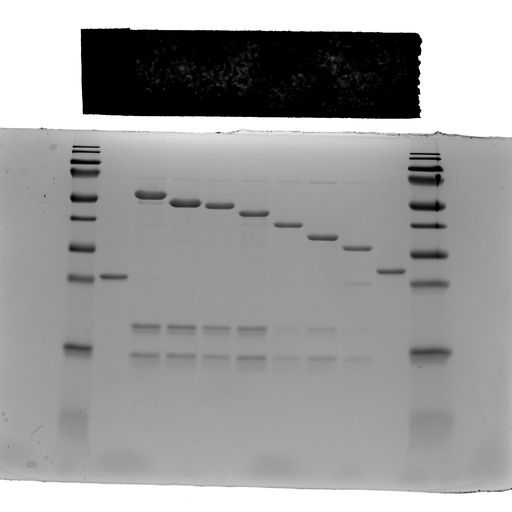

Supplement: Figure 3—figure supplement 2—source data 1. [file elife-87714-fig3-figsupp2-data1.zip › Figure 3-Figure Supplement 2-Source Data 1/Figure 3-Figure Supplement 2B/H2A.Z-H2B pull-down.tif]

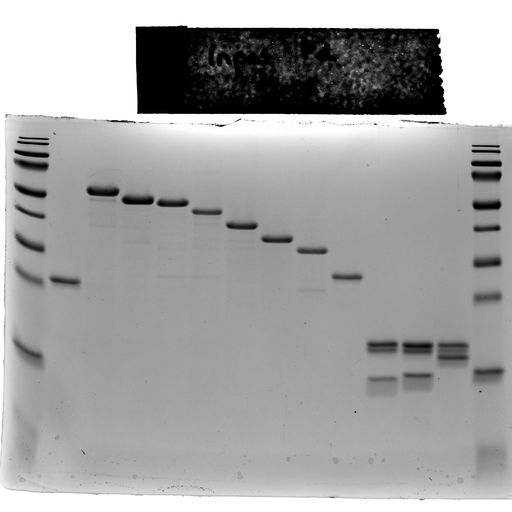

Supplement: Figure 3—figure supplement 2—source data 1. [file elife-87714-fig3-figsupp2-data1.zip › Figure 3-Figure Supplement 2-Source Data 1/Figure 3-Figure Supplement 2B/Input.tif]

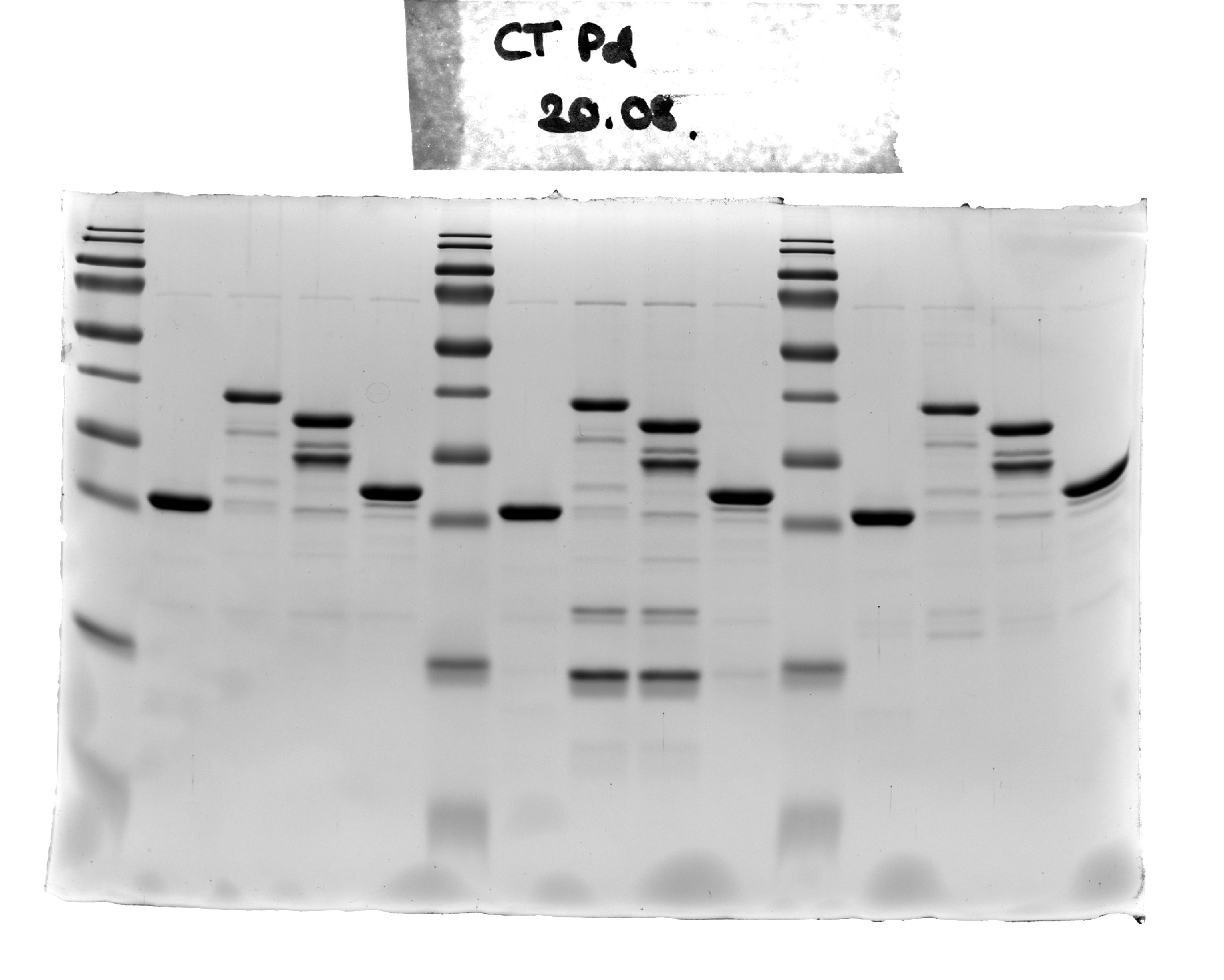

Supplement: Figure 3—figure supplement 2—source data 1. [file elife-87714-fig3-figsupp2-data1.zip › Figure 3-Figure Supplement 2-Source Data 1/Figure 3-Figure Supplement 2C/H2A-H2B H2A.Z-H2B H2A.W-H2B pull-down right panels.tif]

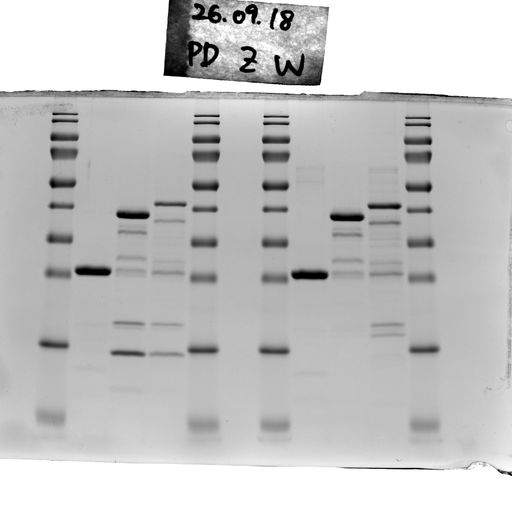

Supplement: Figure 3—figure supplement 2—source data 1. [file elife-87714-fig3-figsupp2-data1.zip › Figure 3-Figure Supplement 2-Source Data 1/Figure 3-Figure Supplement 2C/H2A.Z-H2B H2A.W-H2B pull-down left panels.tif]

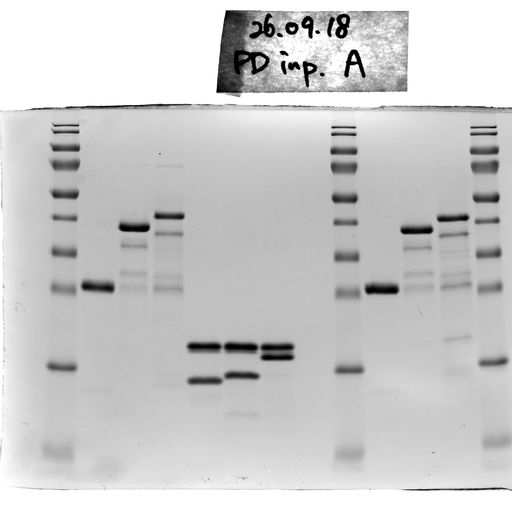

Supplement: Figure 3—figure supplement 2—source data 1. [file elife-87714-fig3-figsupp2-data1.zip › Figure 3-Figure Supplement 2-Source Data 1/Figure 3-Figure Supplement 2C/Input_left panels and H2A-H2B pull-down.tif]

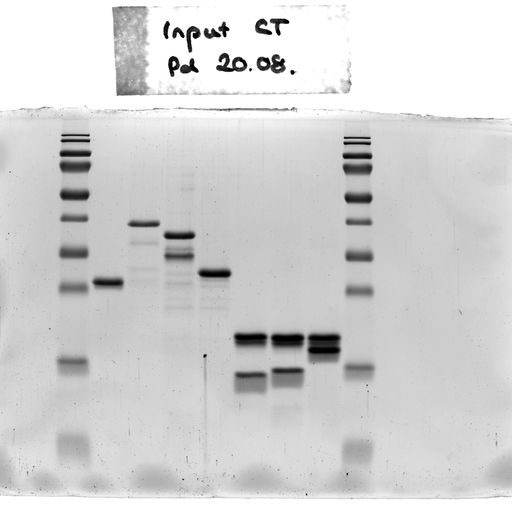

Supplement: Figure 3—figure supplement 2—source data 1. [file elife-87714-fig3-figsupp2-data1.zip › Figure 3-Figure Supplement 2-Source Data 1/Figure 3-Figure Supplement 2C/Input_right panels.tif]
