# Supplementary material for: Histone variants shape chromatin states in Arabidopsis
Source: eLife. 2023 Jul 19;12:RP87714. doi: 10.7554/eLife.87714 (PMC10393023; doi:10.7554/eLife.87714)

Figure 3-Figure Supplement 2A

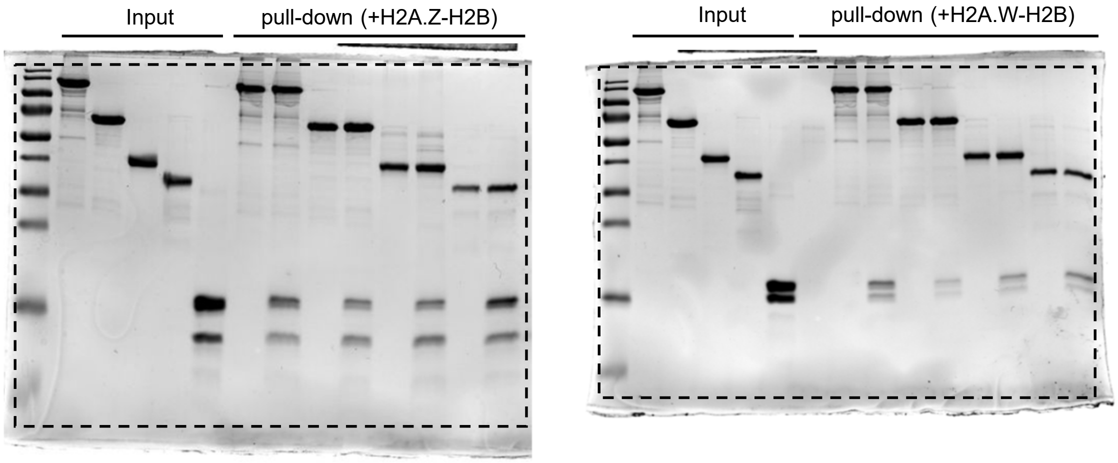

Figure 3-Figure Supplement 2B

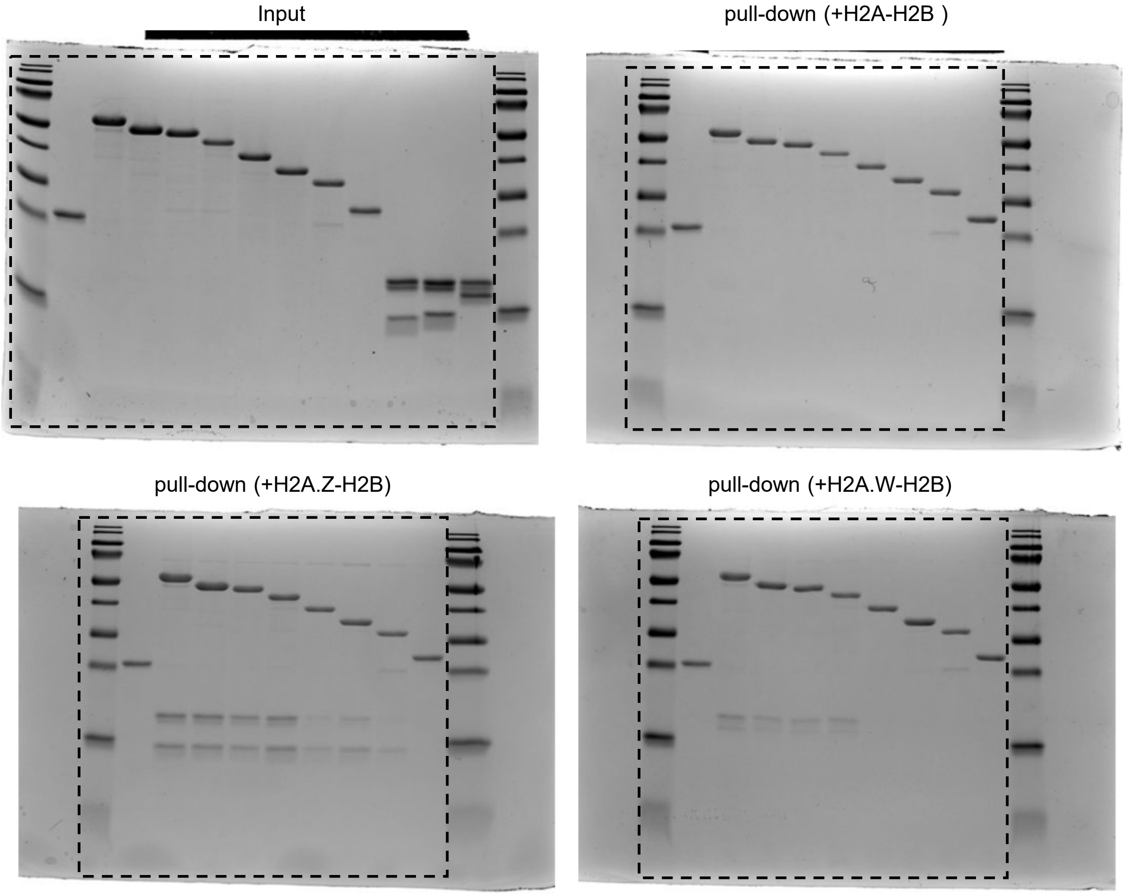

Figure 3-Figure Supplement 2C left panels

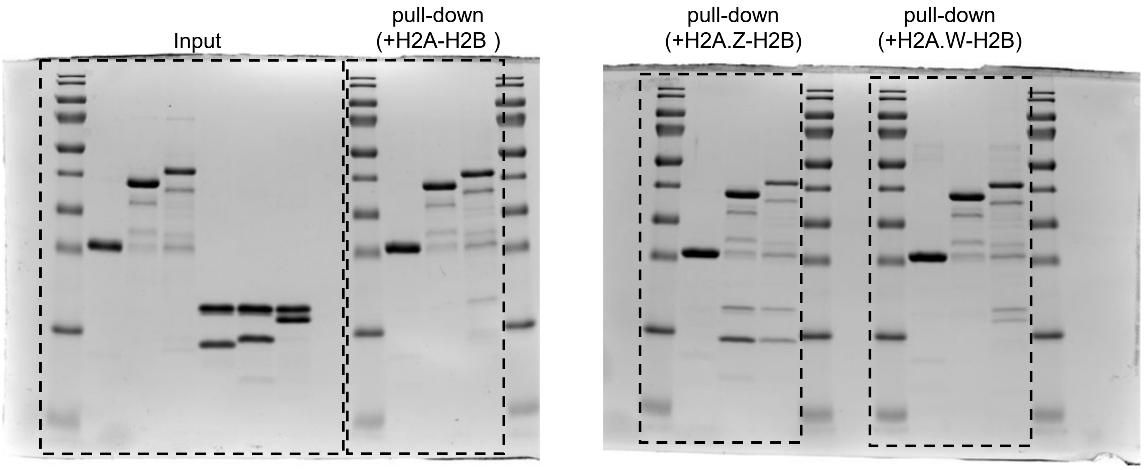

Figure 3-Figure Supplement 2C right panels

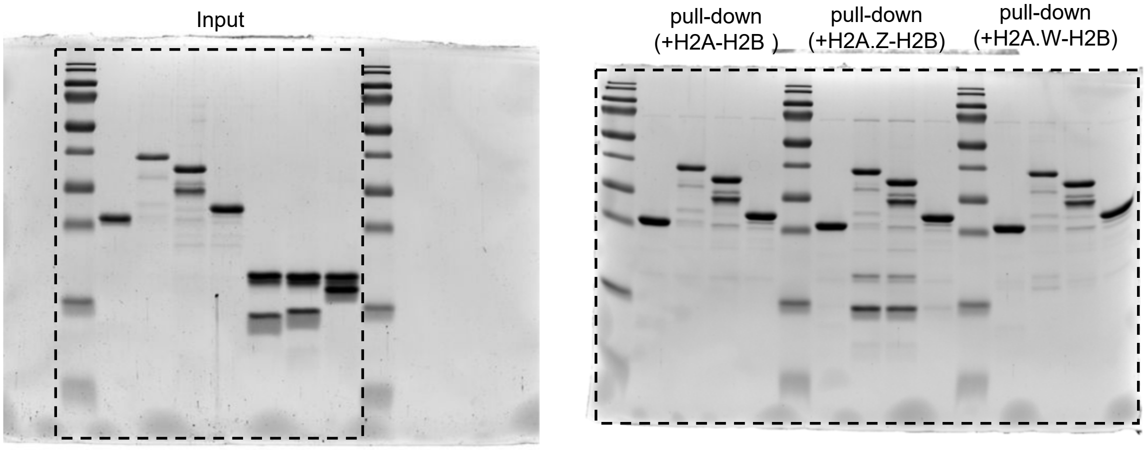

Supplement: Figure 3—figure supplement 2—source data 1. [file elife-87714-fig3-figsupp2-data1.zip › Figure 3-Figure Supplement 2-Source Data 1/Figure 3-Figure Supplement 2A-C.pdf]
